# Single-cell transcriptomics link gene expression signatures to clinicopathological features of gonadotroph and lactotroph PitNET

**DOI:** 10.1186/s12967-024-05821-4

**Published:** 2024-11-15

**Authors:** T. Elise Potthoff, Carolin Walter, Daniela Jeising, Daniel Münter, Archana Verma, Eric Suero Molina, Walter Stummer, Martin Dugas, Wolfgang Hartmann, Matthias Dottermusch, Lea Altendorf, Ulrich Schüller, Sophia Scheuermann, Christian Seitz, Thomas K. Albert, Kornelius Kerl

**Affiliations:** 1 Department of Paediatric Haematology and Oncology, University Children’s Hospital Münster, Albert-Schweitzer-Campus 1, 48149 Münster, Germany; 2 Institute of Medical Informatics, University of Münster, 48149 Münster, Germany; 3 Department of Neurosurgery, University Hospital of Münster, 48149 Münster, Germany; 4 Institute of Medical Informatics, Heidelberg University Hospital, Heidelberg, Germany; 5 Division of Translational Pathology, Gerhard Domagk Institute of Pathology, University Hospital Münster, 48149 Münster, Germany; 6 Institute of Neuropathology, University Medical Center Hamburg-Eppendorf, 20251 Hamburg, Germany; 7 Department of Paediatric Haematology and Oncology, University Medical Center Hamburg-Eppendorf, 20251 Hamburg, Germany; 8 Research Institute Children’s Cancer Center, 20251 Hamburg, Germany; 9 DFG Cluster of Excellence 2180 ‘Image-Guided and Functional Instructed Tumor Therapy’ (iFIT), University of Tübingen, 72076 Tübingen, Germany; 10 Department of Pediatric Hematology and Oncology, University Hospital Tübingen, 72076 Tübingen, Germany; 11 German Cancer Consortium (DKTK) and German Cancer Research Center (DKFZ), Partner Site, Tuebingen, Germany

**Keywords:** Pituitary neuroendocrine tumor (PitNET), Gonadotroph, Lactotroph, Single-cell RNA sequencing, Tumor microenvironment, Intratumoral heterogeneity

## Abstract

**Background:**

Pituitary neuroendocrine tumors (PitNET) are among the most common intracranial tumors. Despite a frequent benign course, aggressive behavior can occur. Tumor behavior is known to be under the influence of the tumor microenvironment (TME). However, the relationship between TME cells and aggressive tumor behavior has not been adequately explored in PitNET.

**Methods:**

We performed differential expression analysis as well as gene expression program identification based on single-cell RNA sequencing to comparatively characterize the transcriptome of seven gonadotroph and three lactotroph PitNET and correlate it with clinical features using bulk RNA-seq data from an independent cohort of 134 PitNET. Tumor immune infiltration was quantified via immunostaining on tissue sections of gonadotroph and lactotroph PitNET.

**Results:**

In lactotroph PitNET, we detect a highly proliferative gene profile with significantly increased expression levels in aggressively growing tumors within bulk RNA-seq data of an independent cohort of 134 PitNET samples. We also report high intratumoral heterogeneity in gonadotroph PitNET (GoPN) and lactotroph PitNET (LaPN) and identify signatures of epithelial, endocrine, and immunological gene networks in both subtypes. A comparison of their TME composition shows enrichment of SPP1^+^ macrophages and CD4^+^ T cells in GoPN, as well as enrichment of CD4/CD8 double-negative T cells (DN) and natural killer cells (NK) in LaPN. Also notable is the presence of proliferative lymphocytes, the occurrence of which positively correlates with more aggressive tumor behavior in the bulk RNA-seq cohort. However, increased CD8^+^ T and NK cell abundances correlate significantly with reduced aggressiveness indicating potential anti-tumoral effects.

**Conclusions:**

Our study expands the knowledge of the differences in cellular composition of gonadotroph and lactotroph PitNET subtypes. It lays the foundation for further studies on the influence of lymphoid cells on the variable aggressive behavior of PitNET. Regarding the treatment of drug-resistant lactotroph PitNET, proliferative lymphocytes, CD8^+^ T, and NK cells could represent potentially valuable targets for developing new cancer immunotherapies.

**Supplementary Information:**

The online version contains supplementary material available at 10.1186/s12967-024-05821-4.

## Background

Pituitary neuroendocrine tumors (PitNET) are common, often clinically benign neoplasms arising from the anterior pituitary gland. Although the mortality is low, PitNET can present with a relevant amount of clinical manifestations due to hormonal hypersecretion and mass effects. PitNET account for 10–15% of all intracranial brain tumors and are the most common tumors in the sellar region [1]. Their prevalence is estimated at around 10% in the general population, although most are clinically inapparent [2]. PitNET originate from the hormone-secreting cell types of the anterior pituitary gland, producing six different hormones: follicle-stimulating hormone, luteinizing hormone, prolactin, growth hormone, thyroid-stimulating hormone and adrenocorticotropic hormone [3]. Currently, histological staining of the hormones mentioned above and the determination of three lineage-specific transcription factors are used for the pathological classification of PitNET according to 2022 WHO criteria [4]. These transcription factors (TFs) include PIT1 for tumors with variable somatotroph, lactotroph, and/or thyrotroph differentiation, SF1 for gonadotroph tumors, and TBX19 for corticotroph tumors. Some tumors are not positive for any of the mentioned hormones or TFs and are thus classified as null-cell PitNET, while some can be plurihormonal.

Furthermore, a clinical distinction is made between functioning and non-functioning PitNET. Functioning PitNET (F-PitNET), representing two-thirds of all PitNET [2], are clinically conspicuous mainly by disturbances of the hormonal balance and are most commonly prolactin-secreting PIT1-lineage tumors [1, 5]. Non-functioning PitNET (NF-PitNET), on the other hand, are primarily diagnosed in the context of mass effect symptoms such as headache and/or visual field disturbances or incidentally during imaging due to unrelated medical procedures and consist mainly of gonadotroph PitNET of the SF-1 lineage [6].

Transsphenoidal surgical resection remains first-line treatment for all PitNET, except lactotroph PitNET [4]. 80–90% of lactotroph PitNET are clinically manageable with dopamine agonists, resulting in reduced tumor size and PRL secretion [4, 5]. However, not all treatment is effective, and recurrency rates in PitNET remain high and hard to predict [7], making their treatment in clinical practice challenging.

The pathophysiological processes involved in PitNET remain poorly understood, particularly regarding the development of more aggressive phenotypes. Therefore, a better understanding of the molecular mechanisms contributing to tumor progression and invasiveness of aggressive PitNET phenotypes is necessary.

Both intratumoral heterogeneity (ITH) and the tumor microenvironment (TME) are increasingly recognized as factors of great importance in the progression and behavior of tumors in general.

The TME of PitNET has been studied increasingly in recent years after seeing significant effects in immunomodulant therapies in other entities [8–10]. It has become clear that PitNET are significantly infiltrated by a variety of immune and stromal cells [11–13], and several studies have aimed to unravel their role in tumor behavior such as proliferation and invasion or response to treatment using a variety of distinct methods such as immunohistochemistry [14, 15], flow cytometry [13], RNA sequencing (RNA-seq) and related technologies [9, 16, 17]. Single-cell RNA sequencing (scRNA-seq) has proven to be an excellent method to explore ITH. Recently, scRNA-seq studies performed on human PitNET have contributed to a deeper understanding of the cellular landscape and biological features of these tumors [9, 17–20]: Zhang et al. found that corticotroph NF-PitNET, compared with functional PitNET, exhibit signs of increased plasticity in both stromal TME and tumor cells and lower signs of active hormone secretion within the latter [17]. Wang et al. found evidence for an association between a higher abundance of stromal cells and increased stiffness of surgically removed PitNET [9]. Furthermore, a new classification of PitNET was proposed by Zhang et al., distinguishing between poor and well differentiated samples within each developmental lineage, based on ITH analyses of 21 PitNET of different subtypes [20].

Despite these studies, it is not yet fully elucidated what influence individual components of the TME have on aggressive tumor behavior in PitNET. Here, we compare gonadotroph tumors of the SF1 lineage (GoPN) as the most common representative of NF-PitNET with lactotroph of the PIT-1 lineage (LaPN) as the most common representative of functional PitNET. The latter often show aggressive growth ([21] and references therein).

## Material and methods

### Patient samples

All ten specimens were obtained from patients who underwent endoscopic endonasal transsphenoidal surgery at the University Hospital of Münster in 2021 with ethical committee agreement (2017-261-f-S, Münster, Germany). Clinical diagnosis was subsequently established by histopathological examination and immunohistochemical staining of the six hormone-specific proteins, namely prolactin (PRL), growth hormone (GH), ß-thyroid stimulating hormone (ß-TSH), adrenocorticotropic hormone (ACTH) and the gonadotropins, ß-luteinizing (ß-LH) and ß-follicle stimulating (ß-FSH) hormone, and three lineage-specific transcription factors pituitary-specific positive transcription factor (PIT-1), steroidogenic factor 1 (SF-1) and T-box transcription factor (T-Pit) at the Institute of Neuropathology, University of Münster. Patients’ clinical data were collected retrospectively from medical records (Suppl. Table 1).

### Tumor sample processing for single-cell experiments

To prepare the single-cell solution for scRNA-seq analysis, fresh tumor samples were manually minced and then enzymatically dissociated for 20–40 min at 37 °C using Papain PDS Kit Component (CellSystems, LK003178) and DNAsel (Worthington, 2139) solved in 5 mL DMEM:F12/HAM (Gibco™ Life Technologies, 11330-057). The enzymatic treatment was accompanied by further mechanical dissociation and stopped by dilution with PBS and further washing steps. According to manufacturer’s instructions, red blood cell lysis was performed using ACK lysis buffer (Thermo Fischer Scientific. A1049201). Cell counting was performed using Trypan Blue Solution (sigma, T8154-100ML), and fluorescence-activated cell sorting (FACS) was used for quality control.

### Single-cell RNA sequencing

Single cells were captured using Chromium Single Cell 3' Gel Bead Kit v2 (10X Genomics) and then loaded as input into the Chromium Controller. Single cell GEMs (gel beads in emulsion) were then generated, followed by GEM-RT, Dyna beads cleanup, cDNA amplification, and SPRIselect beads cleanup. All procedures were performed according to the manufacturer's protocol. Illumina sequencing-ready indexed single-cell libraries were prepared using the Library Bead Kit and Chromium i7 Multiplex Kit. Quality, size, purity, and concentrations of individual cDNAs and corresponding libraries were assessed using Tapestation 2000 (Agilent Technologies). All libraries were sequenced using the NextSeq 500 sequencing platform (high-output kit, 75 cycles v2 chemistry) at the Core Facility (University Hospital of Münster, Münster, Germany).

### Processing of scRNA-seq data

All PitNET scRNA samples were processed with the 10x Genomics CellRanger pipeline v6.0.2 [22], using the human transcriptome GRCh38 v2020-A as reference for CellRanger's count function and default values otherwise. Further analyses were conducted in R, version 4.0.5 [23], and with the R package Seurat v4.0.5 [24].

### Quality control and further processing

Seurat objects were created separately for each PitNET sample, and only cells with at least 200 features and less than 25% of mitochondrial genes were kept for further analyses. In addition, only features present in at least three cells were kept for each sample. The PitNET data was then split into subsets for the GoPN (n = 7) and LaPN (n = 3) subtypes. For each subset all samples were log-normalized, integrated, and clustered with the essential Seurat functions using a resolution parameter of 0.5 for the clustering, and default values otherwise. CellRanger's force-cells option was set to 9500 for sample KK21-H-136 due to issues with the default cell detection.

### Differential expression (DE) analysis and gene expression program annotation

Seurat's feature plots were then used to create UMAP plots for chosen genes and gene signatures, and the clusters were annotated based on the expression of target genes, and differentially expressed genes per cluster. All clusters with multiple separated cell populations were subsequently subclustered with Seurat functions. Additionally, native Seurat functions were used to create dot plots, while heatmaps were visualized with the R/Bioconductor package pheatmap [25]. The relative contribution of samples to each cluster was visualized using ggplot2's stacked bar graphs [26].

Finally, candidate regions for copy-number variations (CNVs) were identified based on their relative over- or underexpression of related genes with the R package CONICSmat [27], and heatmaps indicating these expression variations were plotted with CONICSmat functions. Tumor clusters for both PitNET datasets were classified based on the expression of specific marker genes and the presence or absence of inferred CNV candidates.

### Processing bulk RNA-seq data

The mRNA-seq dataset on ArrayExpress (#E-MTAB-7768) associated with the Neou et al. publication [28] was downloaded, and the mRNA-seq read counts were DESeq2 normalized and log2-transformed. The expression levels of distinct single-cell signatures established in our work were added and linearly transformed to the interval [0, 1] to create signature scores in the bulk expression data and then plotted in BoxPlots using ggplot2. Statistical information was obtained using unpaired Wilcoxon test, with */**/***/**** indicating *p* ≤ 0.05/0.01/0.001/0.0001, respectively and can be found in Suppl. Table 4, 5.

### Immunostaining of human PitNET

The level of tumor immune infiltration in gonadotroph and lactotroph PitNET was quantified by staining of tissue sections (GoPN: n = 20; LaPN: n = 19) with the established myeloid marker *CD68* and the T cell marker *CD3*. Subsequently, scoring was performed via independent and blinded examination by two experienced pathologists. The Mann–Whitney U test was used to determine statistical relevance.

## Results

### Transcriptomic landscapes of functioning and non-functioning PitNET

**Fig. 1:**
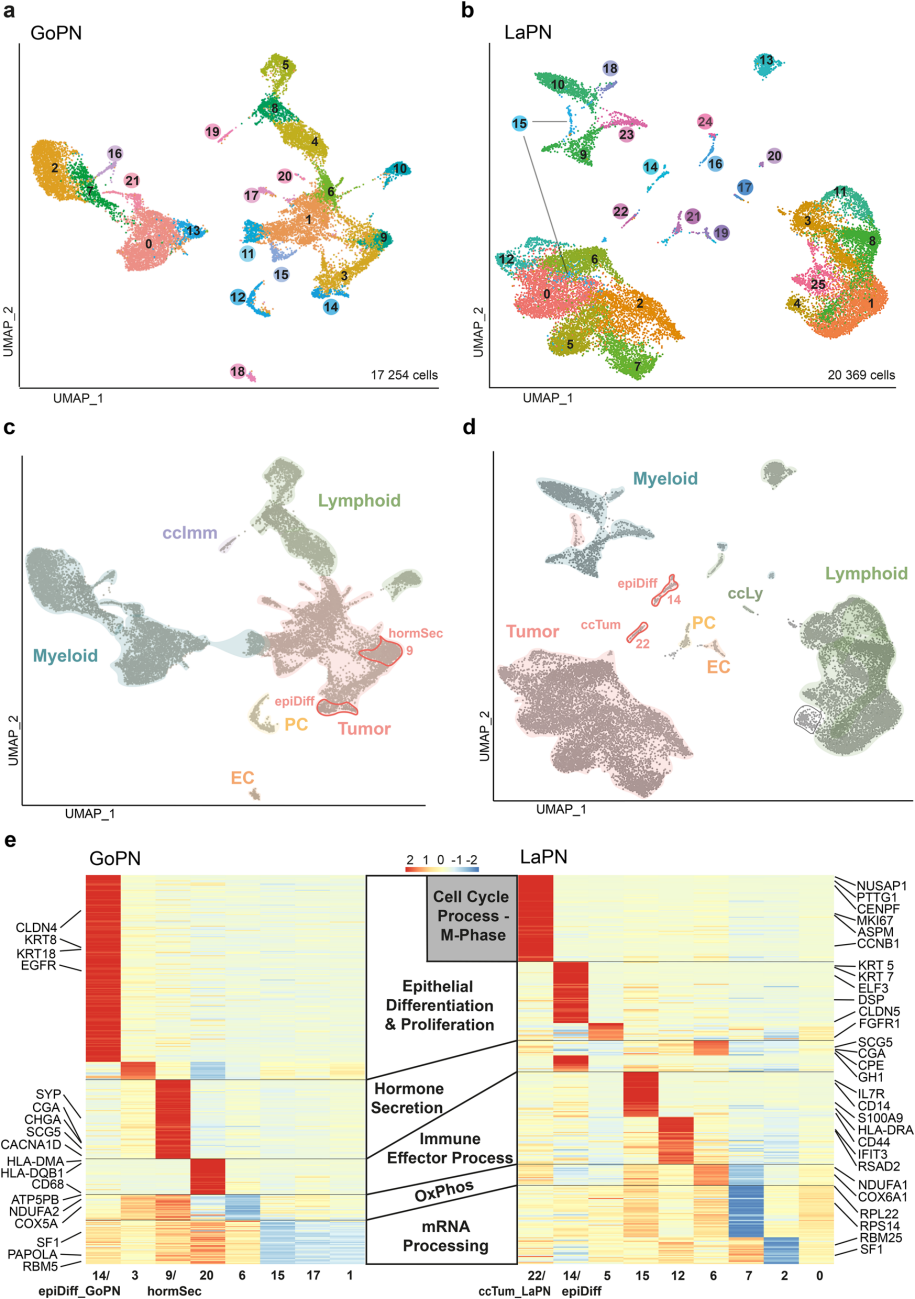
The single-cell transcriptomic landscapes of GoPN and LaPN. **a** UMAP plot showing integration and clustering of seven GoPN samples and **b** of three LaPN samples, as determined by scRNA-seq. **c** Cell type annotation of GoPN, and **d** of LaPN based on known marker genes and inferred CNV analysis (see Suppl. Figure 2 and Suppl. Figure 3). Clusters highlighted in red indicate selected tumor cell clusters (cluster 9 and 14 in GoPN, cluster 14 and 22 in LaPN) with specific gene signatures. Heatmaps of the relative expression levels of these and other tumor cell cluster-specific gene signatures are shown in (**e**), together with their functional gene ontology association and representative marker genes. *ccImm* cycling immune cells, *ccTum* cycling tumor cells, *EC* endothelial cells, *epiDiff* epithelial differentiation and proliferation, *GoPN* gonadotroph PitNET, *hormSec* hormone secretion, *OxPhos* oxidative phosphorylation, *LaPN* lactotroph PitNET, *PC* pericytes, *UMAP* uniform manifold approximation and projection

We performed scRNA-seq of postoperative tissue from primary tumors of seven untreated GoPN and three untreated LaPN patients (see Methods for details; Suppl. Table 1 for clinical data). Subsequent UMAP embedding of 17,254 single-cell transcriptomes for the integrated GoPN samples revealed 21 clusters and that of the 20,369 single-cell transcriptomes of the LaPN samples revealed 25 clusters (Fig. 1a, b; Suppl. Figure 1a, b; Suppl. Table 2). Then, using specific marker genes and matching with reference datasets, we first annotated the definite TME clusters and then confirmed the potential tumor cell clusters by detecting deviant profiles in inferred copy number variation (CNV) (Fig. 1c, d; Suppl. Figure 2, 3).

### Intratumoral heterogeneity in GoPN and LaPN

Next, we focused on the characterization of the intratumoral heterogeneity (ITH) in GoPN and LaPN. Using differentially expressed gene (DEG) analysis, specific gene sets were identified for each individual cluster (Suppl. Table 3). These were then functionally annotated via Gene Ontology (GO) and presented in a heatmap to visualize the differences and similarities of different tumor cell subpopulations within GoPN and LaPN (Fig. 1e). In both subtypes, there are cell populations that behave relatively inertly concerning specific processes. These are processes where basic housekeeping or metabolic processes such as oxidative phosphorylation and mRNA processing are down-regulated (GoPN: cluster 1, 6, 15, 17; LaPN: cluster 0, 2, 7). In addition to these rather inconspicuous cell populations, there are also those whose gene expression signature indicate the participation in highly specific biological processes such as epithelial differentiation, hormone secretion or immune effector processes. However, it is essential to note that the respective signature genes for GoPN and LaPN are distinct despite these functional similarities. For example, the signature “epithelial differentiation and proliferation” (epiDiff) contains different representatives of the keratin gene family (GoPN: *KRT8*, *KRT18*; LaPN: *KRT5*, *KRT7*), the claudin gene family (GoPN: *CLDN4*; LaPN: *CLDN5*) or the growth factor receptor gene family (GoPN: *EGFR*; LaPN: *FGFR1*) in the two subtypes. Another signature is functionally associated with "hormone secretion" (hormSec), which indicates these tumor cells' endocrine origin. In this case, there is more overlap between the respective signatures: Both that of GoPN and LaPN contain the hormone gene *CGA* and the neuroendocrine marker genes chromogranin A (*CHGA*), secretogranin V (*SCG5*), and carboxypeptidase E (*CPE*). Further, a signature associated with “immune effector processes” can be detected in both GoPN and LaPN. Multiple genes related to immune cell activation and chemotaxis are contained in both signatures, e.g., in GoPN *HLA-DMA, HLA-DQB1, and CD68*, or LaPN *HLA-DRA, S100A9, IL7R, CD14, and CD44*. In addition, the LaPN signature contains genes related to antiviral processes, such as *IFIT3* and *RSAD2* (Fig. 1e).

Finally, this analysis revealed a small LaPN-restricted tumor cell population in cluster 22 (Fig. 1d, e). Its signature is characterized by a significant upregulation of numerous cell cycle and proliferation-associated genes such as cyclin B1 (*CCNB1*), centromere protein F (*CENPF*), or the proliferation marker Ki-67 (*MKI67*). Also included is the human securin gene *PTTG1*, which was initially identified as an oncogene in malignant pituitary tumors but has been associated with tumor aggressiveness and invasiveness in various other cancer entities [29]. In the following, this LaPN-specific signature is referred to as "cycling tumor cells" or ccTum. Notably, a related tumor cell population is missing in the non-functioning GoPN subtype.

In summary, both GoPN and LaPN exhibit high intratumoral heterogeneity. In both subtypes, there are similarities in functional networks within epithelial, endocrine, or immunological gene expression programs, but also a tumor cell population with highly proliferative characteristics that is exclusively present in LaPN.

### Clinicopathological relevance of GoPN and LaPN single-cell signatures

Next, we investigated whether and how these signatures correlate with the clinicopathological properties of these tumors. To this end, we first checked whether these signatures can also be found in a large validation cohort, in which a total of 134 tumor transcriptomes from eight WHO-classified PitNET subtypes—including GoPN, LaPN, somatotroph (SomPN), thyrotroph (ThyPN), and corticotroph (CorPN)—have been analyzed using bulk RNA-seq [28]. For this comparison, we developed a scoring procedure in which the reference data of the validation cohort received a so-called signature score between 0 (no agreement) and 1 (complete agreement) (see Methods for details; Suppl. Tables 4, 5).

**Fig. 2:**
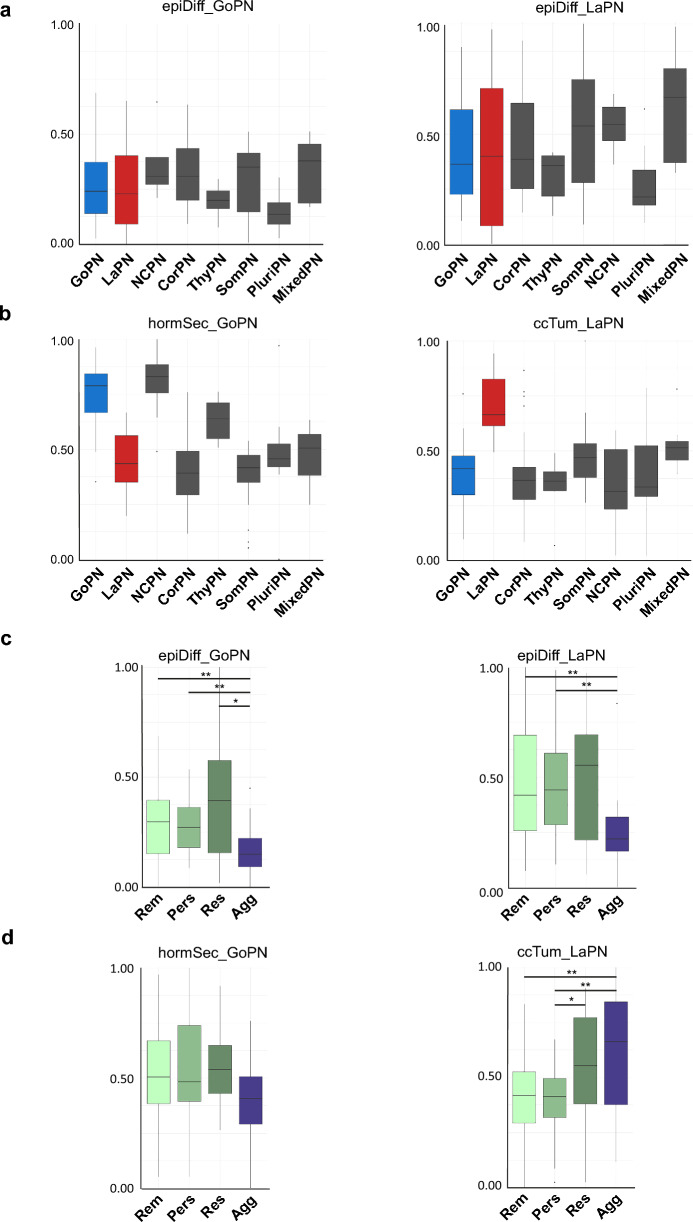
Tumor-specific gene signatures correlate with clinicopathological features of PitNET. **a** Boxplots showing the normalized expression level of the condensed epithelial gene signatures derived from GoPN or LaPN single-cell data (*epiDiff_GoPN*, *epiDiff_LaPN*) in a bulk RNA-seq cohort comprising 134 PitNET tumors (29 gonadotroph (GoPN), 16 lactotroph (LaPN), 8 null-cell (NCPN), 35 corticotroph (CorPN), 6 thyrotroph (ThyPN), 23 somatotroph (SomPN), 9 plurihormonal (PluriPN) and 8 mixed (MixedPN) PitNET) [28]. **b** Boxplots of the normalized expression levels of the condensed hormone secretion signature (*hormSec*) from GoPN (left) and the cycling tumor cell signature (*ccTum*) from LaPN (right). For a statistical analysis of (**a**, **b**) see Suppl. Table 5. **c**, **d** Correlation of GoPN or LaPN gene signatures with aggressiveness level and/or clinical behavior of the bulk RNA-seq PitNET cohort (*Rem*, remission, n = 60; *Pers*, persistent, n = 48; *Res*, resistant, n = 12; *Agg*, aggressive, n = 14). Statistical analysis was performed using an unpaired two-sample Wilcoxon test, with */**/***/**** indicating *p* ≤ 0.05/0.01/0.001/0.0001, respectively. Non-significant results remain unmarked

Essentially all signatures derived from our single-cell analysis were found in the bulk RNA-seq data of the different PitNET subtypes, underlining the validity and relevance of our approach (Fig. 2). However, very different scores were sometimes obtained for individual signatures within the respective subtypes. For example, while the two epithelial signatures epiDiff_GoPN and epiDiff_LaPN showed an essentially uniform distribution of their score (Fig. 2a), the values for the endocrine signature hormSec_GoPN and the proliferation-associated signature ccTum_LaPN were distributed much more specifically. It is noticeable that, in addition to GoPN itself, hormSec_GoPN has the relatively highest score in null-cell PitNET (NCPN), i.e. another representative of non-functioning PitNET (Fig. 2b). Even more striking was the specificity of ccTum_LaPN, which showed an average expression level in lactotroph PitNET that was about twice as high as in all other subtypes (Fig. 2b).

In addition to their WHO classification and global gene expression profiles, other relevant data, such as the patient's response to treatment, are available for the samples of the reference cohort. Based on the clinical course, each sample is classified into one of four categories, in ascending order of severity from „remission” (*Rem*) to „persistent” (*Pers*) to „resistant” (*Res*) to „aggressive” (*Agg*) [28]. The definitions are as follows: (i) Classified as *Agg* are tumors that required unusual treatments (e.g., multiple surgeries, radiation therapy, or chemotherapy) and/or tumors that showed resistance to first-line treatments. (ii) *Res* refers to resistant tumors that grew slowly but did not require radiation or chemotherapy. (iii) *Pers* refers to those defined by stable disease or slow tumor growth after incomplete surgery, and (iv) *Rem* includes all tumors in remission after first-line therapy. Looking more closely at the PitNET subtypes we examined, the lactotroph PitNET samples stand out with an increased aggressive phenotype, along with thyrotroph, corticotroph, and plurihormonal PitNET. One quarter of the 16 lactotroph PitNET samples fall into the category *Agg*. In contrast, the gonadotrophic PitNET samples (n = 29) show much less aggressive features and are found in either one of the two less aggressive categories *Rem* or *Pers* (Suppl. Figure 4).

When comparing our sc-derived signatures with these clinical data, it is noticeable that the scores of all four signatures gradually increase from category *Pers* to *Res* (Fig. 2c, d). However, while in the case of the epithelial and endocrine signatures, the scores in aggressive tumors drop again, this value increases significantly in the case of the proliferation signature ccTum_LaPN (Fig. 2d).

In summary, we used scRNA-seq to identify a tumor cell population mainly present in the F-PitNET subtype LaPN and characterized by a gene expression signature associated with a more resilient and aggressive clinical presentation.

### The tumor microenvironment of GoPN and LaPN

Previous single-cell studies of PitNET have not, or only sparsely, addressed the composition of the TME and its potential impact on tumor growth or other pathological processes [18, 20]. However, our study shows that GoPN and LaPN represent two entities harboring a comparatively rich TME (Fig. 1c, d) compared to PitNET of the TPIT lineage [17]. As the TME is assumed to impact pituitary tumor behavior significantly [12, 30], we have focused on a closer examination of this aspect in the following.

**Fig. 3:**
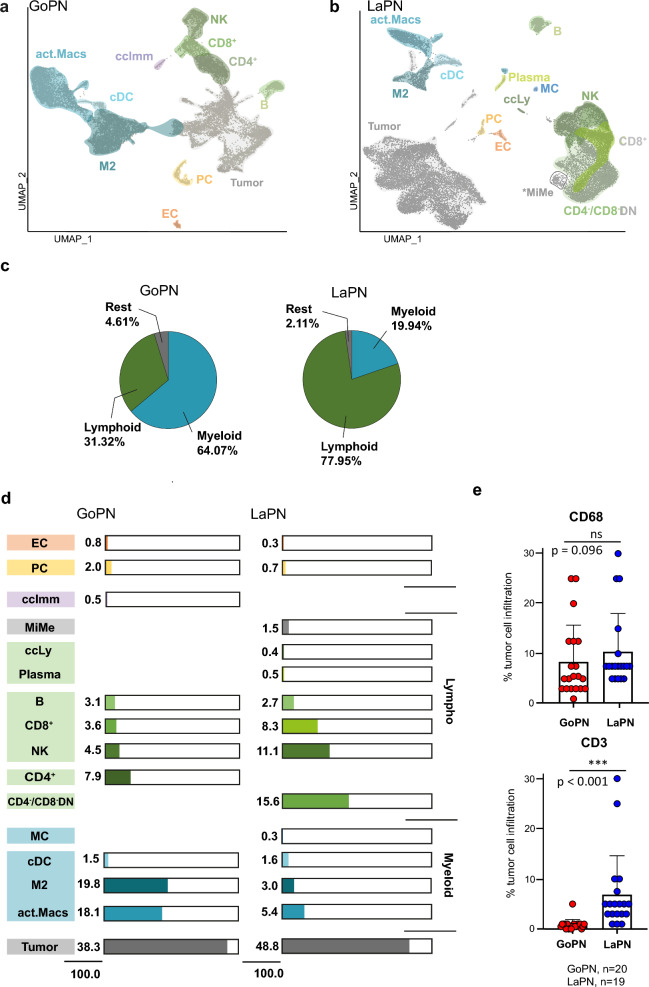
Tumor microenvironment of GoPN and LaPN. **a** UMAP plot with cell type annotation of GoPN, and **b** of LaPN tumors. **c** Pie charts showing the relative proportions of lymphoid, myeloid, and other cell types in the TME of GoPN and LaPN. **d** Relative distribution of all cell types, including tumor cells in GoPN (left) and LaPN (right), based on the number of cells per annotated cluster. **e** Quantification of immunostaining of the myeloid marker CD68 (top) and the T cell marker CD3 (bottom) in tissue sections of GoPN (n = 20) and LaPN (n = 19) tumors. Relative tumor cell infiltration (y-axis) was assessed by expert judgment, and the differences in mean score values of GoPN (red) versus LaPN (blue) were tested for significance using a Mann–Whitney U-test. ns, not specific. *B* B-cells, *ccLy* cycling lymphoid cells, *ccImm* cycling immune cells, *CD4*^−^/*CD8*^−^*DN* CD3^+^CD4/CD8-double negative T cells, c*DC* *CD1C*-expressing conventional dendritic cells, *EC* endothelial cells, *act.Macs* activated macrophages (act-Macs), *MC* mast cells, *MiMe* high-level expression of mitochondrial and metabolic genes, *N*K natural killer cells, *PC* pericytes, *Plasma* plasma B-cells

An initial mapping of the cellular TME landscape is shown in Fig. 3a for GoPN and Fig. 3b for LaPN. With few exceptions, both subtypes show similar distributions of the individual cell types between the respective samples (Suppl. Figure 5). TME cells represented more than half of all cells analyzed, namely 61.7% in GoPN and 51.2% in LaPN. Within the TME, immune cells dominated with proportions of approximately 95% and 98%, respectively (Fig. 3c, d). Although we were also able to detect pericytes (PC) and endothelial cells (EC) (Suppl. Figure 2), we refrained from further analyzing them due to their small number (GoPN: 349 PC, 142 EC; LaPN: 149 PC, 71 EC) and low proportion (GoPN: 4.6%; LaPN: 2.1%; Fig. 3c). This first overview of the immune cell landscape already illustrates a striking difference between GoPN and LaPN, namely the TME dominated by myeloid cell types in GoPN (64% myeloid vs. 31% lymphoid cell types) and, vice versa, dominance of lymphoid cells in LaPN (78% vs. 20% myeloid cell types) (Fig. 3c; Suppl. Table 6).

A closer look revealed other distinct features of the immune cell landscape. We found small subpopulations of proliferating immune cells (ccImm) in GoPN and proliferating lymphoid cells (ccLy) in LaPN. In the latter, we were able to detect small populations of mast cells (MC) and plasma B cells (Plasma), both of which were absent in GoPN. Moreover, we also observed a cell group that—although clustering together with other lymphoid cell types—did not express any corresponding marker genes but was characterized by a high expression of mitotic and metabolic genes and is referred to here as "MiMe" (Fig. 3b, d).

Upon closer examination of the myeloid compartment, we find activated and M2-polarized macrophages (Macs) in the TME of subtypes. Expression of the M2-specific markers *CD163* and *MRC1/CD206* [31, 32] is found in cluster 9 (LaPN) as well as clusters 0, 11 and 13 (GoPN) (Fig. 3a, b). M2-Macs often possess pro-tumorigenic effects and have been associated with higher invasion in PitNET [15, 33].

Next, we observed myeloid cells with increased *VCAN, S100A8/S100A9* and *IL1B* expression indicating that these cells are active in inflammatory processes (Suppl. Figure 2). In the following, we refer to them as activated macrophages (act-Macs). Interleukin 1 beta (*IL1B*) plays an essential role in the induction of inflammatory and anti-tumoral activities [34]. In contrast, elevated *VCAN* and *S100A8/S100A9* expression has been associated with the recruitment of myeloid-derived suppressor cells (MDSC) and, thus, the creation of an immunosuppressive TME in some tumors [35–37]. While act-Macs express *CD44* and *CCR2*, M2-polarized and other macrophages (GoPN: 21; LaPN: cl. 18 and 23) show high-level expression of *CX3CR1*. This expression pattern suggests that act-Macs represent tumor-infiltrating blood-derived macrophages, while the latter are tissue-resident cells [38, 39].

Also, there is a striking difference between the two subtypes in the distribution of T cells and natural killer (NK) cells. For instance, at 11.1%, the latter cell type is almost 2.5 times more abundant in LaPN than in GoPN, where NK cells account for only 4.5% of all cells (Fig. 3d). Moreover, while GoPN is characterized by a comparatively high number of double CD3/CD4-positive (CD4^+^) T helper cells, this cell type is absent in LaPN. In contrast, CD3^+^CD4/CD8-double negative (CD4^−^/CD8^−^DN) T cells, which normally comprise a rare subset of peripheral T cells [40], forms the largest fraction of all non-tumor cells in LaPN at almost 16%, while it is missing in GoPN (Fig. 3d).

We validated this differential immune cell by performing immunostaining on tissue sections from GoPN and LaPN tumors which confirmed significantly stronger lymphoid tumor infiltration in LaPN vs. GoPN (Fig. 3e).

### Differences in the distribution of specific TAM populations

**Fig. 4:**
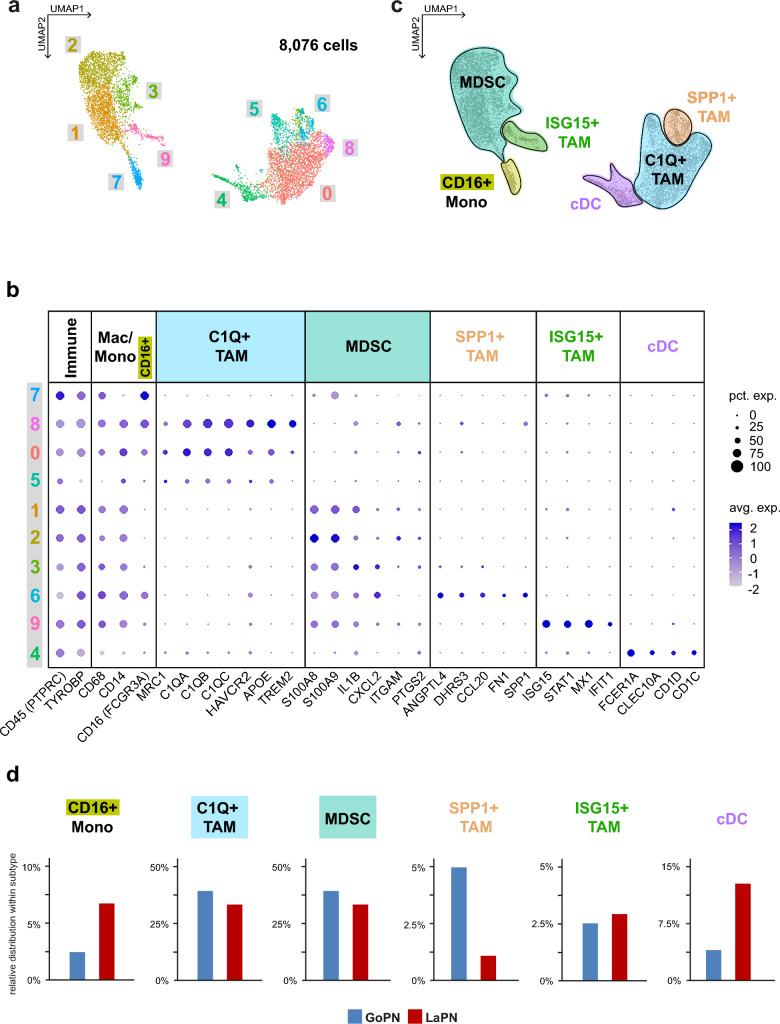
Distinct cell type expressions in myeloid cells in GoPN and LaPN. **a** Cluster arrangement of the myeloid cells from both GoPN and LaPN tumors after integration. **b** Dot plot showing the average expression levels (avg. exp.) and occurrence (pct. exp.) of myeloid cell type-specific marker genes in the individual clusters. **c** UMAP plot with annotated myeloid cells from GoPN and LaPN. **d** Relative distribution of identified cell types within GoPN and LaPN cells. *cDC* *CD1C*-expressing conventional dendritic cells, *MDSC* myeloid-derived suppressor cells, *Mono* monocytes, *TAMs* tumor-associated macrophages

To gain more detailed insights into the different cellular composition of the myeloid compartment of the two subtypes, we isolated the respective myeloid cell clusters from GoPN and LaPN, merged them and re-clustered the integrated myeloid subset of 8,076 cells (Fig. 4a; Suppl. Figure 6a).

This higher resolution of the myeloid compartment and the subsequent comparison with a reference data set of so-called tumor-associated macrophages (TAMs) [41] led to the identification of ISG15^+^, C1Q^+^ and SPP1^+^ TAM populations (Fig. 4b, c; Suppl. Figure 6b). ISG15^+^ and C1Q^+^ TAMs have been previously detected in all three PitNET lineages, while this is notably not the case for SPP1^+^ population [42]. Furthermore, it is striking that the relative frequencies of SPP1^+^ TAMs differ significantly: GoPN (4.9%) contains proportionally about 4.5 times more of these cells than LaPN (1%). This is remarkable as the abundance of SPP1^+^ TAMs is associated with poor prognosis in lung adenocarcinoma and cervical cancer patients [43, 44] as well as the promotion of cancer stemness in pancreatic cancer [45]. Nevertheless, in PitNET we find a notably higher number of SPP1^+^ TAMs in the GoPN cohort, which is associated with significantly better clinical outcomes compared to LaPN.

### The influence of lymphoid cell subsets on the clinical features of PitNET

As shown above the striking difference between the GoPN and LaPN TME lies in the abundance of T cells and natural killer (NK) cells (Fig. 3e). To decipher these differences more precisely, we proceeded as before and generated a subset of all lymphoid cells.

**Fig. 5:**
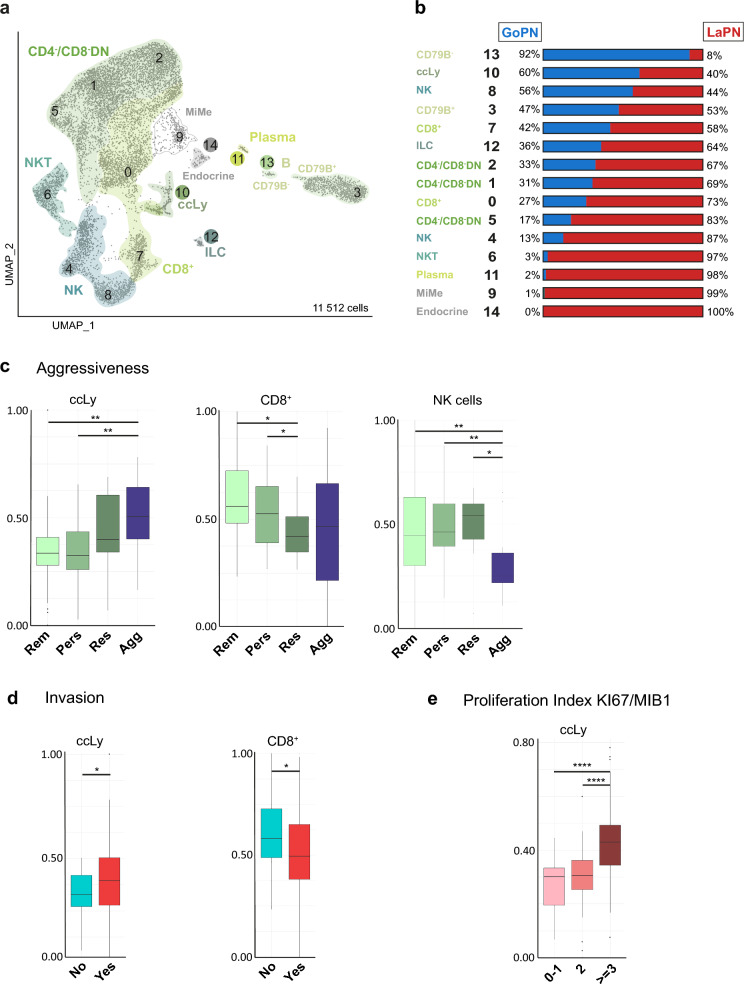
The lymphoid compartment and its relation to clinicopathological features of PitNET. **a** Cluster arrangement of the lymphoid compartment from both GoPN and LaPN tumors after integration. **b** Relative proportions of GoPN and LaPN cells in the identified clusters. **c**–**e** Expression of cycling lymphoid cell (*ccLy*), CD8^+^ T cell (*CD8*^+^), and natural killer cell (*NK*) signatures in PitNET tumors [28], categorized by degree of aggressiveness in (**c**), invasion into the sphenoid sinus in (**d**), or by proliferation index (percentage of MIB1-stained Ki67-positive cells) in (**e**). Statistical analysis was done as in Fig. 2 (Suppl. Table 9). *ILC* innate lymphoid cells

Single-cell transcriptomes of all cells of lymphoid origin from both entities (a total of 11,512 cells) were integrated into a single UMAP and re-annotated (Fig. 5a; Suppl. Figure 7b; Suppl. Table 7). The relative GoPN/ LaPN proportions of each cell type are shown in Fig. 5b, and those of individual samples in Suppl. Figure 7a. As previously observed with the myeloid subset, the higher resolution allows the identification of previously obscured cell types. For example, in addition to a large cluster of CD79B^+^- B cells, we identified a smaller population of CD79B^−^—B cells in cluster 13. Also, a small number (n = 55) of innate lymphoid cells (ILC) now appear in cluster 12. The same applies to natural killer T cells (NKT), which are identified in cluster 6.

After cell type annotation, we derived specific gene signatures for individual, defined subgroups of lymphoid cells as described above for the tumor cells. In the next step, these lymphoid-specific signatures were then correlated with the clinicopathological properties of PitNET by comparison with the RNA-seq validation cohort (Suppl. Tables 8, 9). Starting with the levels of tumor aggressiveness, it was noticed that increasing expression of the proliferative ccLy signature was associated with increased tumor aggressiveness and resistance to treatment, as it was already the case with the proliferative ccTum_LaPN signature. Furthermore, the analysis also shows reduced resistance to treatment coinciding with increased CD8^+^ T cell infiltration as well as a reduced aggressiveness in correlation to a higher amount of NK cells (Fig. 5c). All other lymphoid signatures showed insignificant changes in tumor aggressiveness (Suppl. Figure 8a).

A similar picture is seen in the correlation of expression levels of ccLy and CD8^+^ signatures to tumor invasion data in the sphenoid sinus. Increased ccLy expression is associated with increased invasion, whereas increased CD8^+^ expression is associated with decreased invasion (Fig. 5d). This result complements a publication on somatotroph PitNET, in which both tumors without cavernous sinus invasion and first-generation somatostatin analogs responder tumors showed higher numbers of CD8^+^ lymphocytes compared to invasive or resistant tumors [46].

Finally, the expression levels of ccLy increase significantly with a higher MIB1/KI67 proliferation index of the tumor (Fig. 5e).

### The correlation of tumor and lymphoid cell type signatures regarding their association with aggressive phenotypes

In both the TME and the tumor cells of gonadotroph and lactotroph PitNET, we found clear signatures with expression patterns correlating with either a better outcome (less aggressiveness, less resistance to treatment and/or lower invasiveness), namely epiDiff_GoPN, epiDiff_LaPN, CD8^+^ T cells and NK cells, or a worse outcome (higher aggressiveness, higher resistance and/ or more invasiveness), namely ccTum_LaPN and ccLy. Here, the question arises whether there is a linear relationship between the expression of these distinct signatures. Therefore, we computed a Pearson correlation coefficient between the tumor signatures ccTum_LAPN, epiDiff_GoPN, and epiDiff_LaPN and the lymphoid cell signatures ccLy, CD8^+^, and NK.

**Fig. 6:**
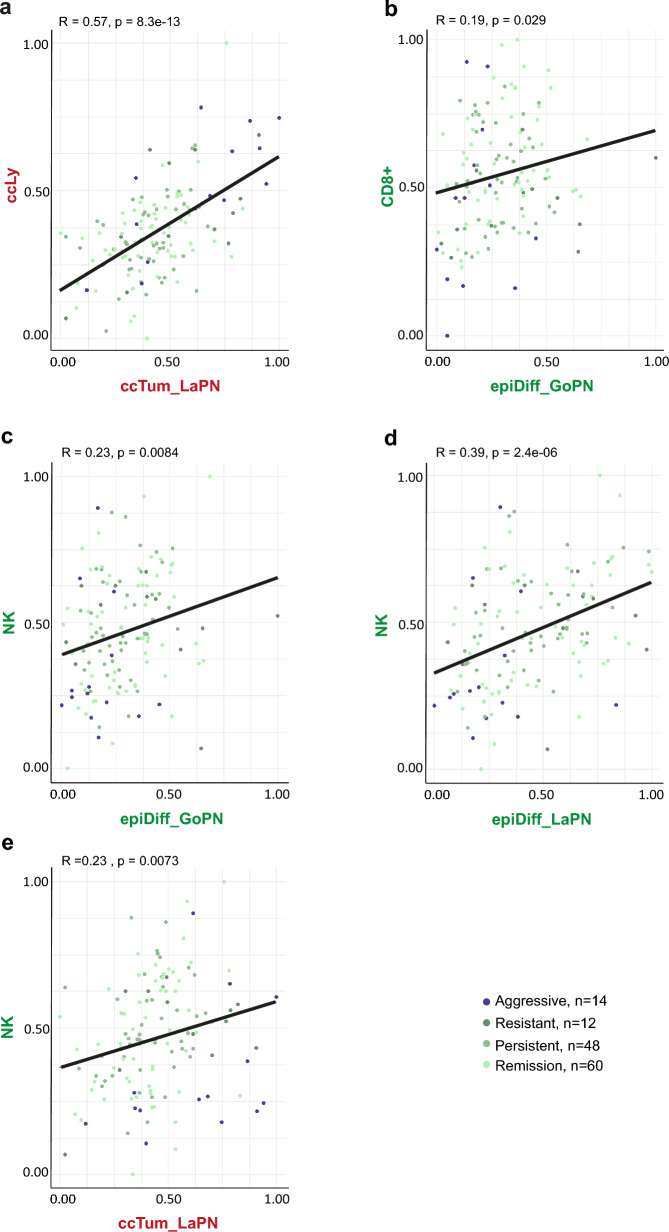
Correlation of distinct tumor and lymphoid cell signatures associated with better or worse tumor progression outcome. Linear correlation of expression values of **a** CD8^+^ T cell (*CD8*^+^) signature with tumor signature *epiDiff_GoPN*, **b** cycling lymphoid cell (*ccLy*) signature with cycling tumor cell (ccTum) signature and natural killer cell (*NK*) signature with both tumor signatures *epiDiff_GoPN* (**c**) and *epiDiff_LaPN* (**d**). Signatures associated with a less aggressive outcome are marked in green, while those associated with a more aggressive outcome are marked in red. Pearson correlation coefficient (R) and p-value are indicated (p)

Interestingly, there is a strong positive correlation (R = 0.57) between the expression of the ccLy signature of the TME and the ccTum_LaPN signature of the LaPN tumor cells (Fig. 6a). Both signatures are associated with higher levels of aggressiveness and invasion in the sphenoid sinus (Figs. 2d and Fig. 5c, d).

The expression of both the CD8^+^ T cell signature and the NK cell signature correlate positively with the expression of the epiDiff signatures of GoPN and LaPN (Fig. 6b–d). All of these signatures are associated with a better outcome. In one case, there is a positive correlation between the expression of a signature associated with a better outcome and one with a worse outcome, NK cells with ccTum_LaPN (Fig. 6e).

## Discussion

In this study, we add to the knowledge of the differences in the cellular composition of gonadotroph and lactotroph PitNET and identify proliferative cells within lactotroph PitNET tumor cells correlating with high aggressiveness in a larger cohort. Furthermore, we provide a detailed overview of the lymphoid cell compartment within the gonadotroph and lactotroph PitNET TME, identifying proliferative lymphocytes with a strong correlation to aggressive and invasive behavior and pointing out potential targets for cancer immunotherapy within the lymphoid compartment.

A comprehensive pan-cancer study has recently shown striking overlap at the ITH level within tumors from different tissues and cell origins [47]. Some of our generated ITH signatures from GoPN and LaPN can be clearly assigned to certain metaprograms (MP) defined by Gavish et al. For example, several genes belonging to the major histocompatibility complex (MHC), such as *HLA-DRA*, *HLA-DMA*, *HLA-DQB1,* but also *IFIT3*, can be found in our immune effector process signatures from GoPN and LaPN, but also in MP17 and MP18 (interferon and MHC-II) defined by the authors. In addition, specific genes from the ccTum_LaPN signature (*NUSAP1, CENPF, CCNB1, PTTG1, MKI67,* and *ASPM*) are part of the defined MP1 (Cell Cycle—G2/M).

Within PitNET, ITH is the subject of ongoing research. Recently, an extensive publication has been published on ITH with many PitNET subtypes based on scRNAseq analysis [20]. We focus on gonadotroph and lactotroph PitNET and identify certain similarities.

In both subtypes, we observe cell populations with distinct epithelial and proliferative expression profiles (epiDiff_GoPN and epiDiff_LaPN) with up-regulated genes from the keratin and claudin family showing strong similarities to the cells described as tumor-stem-like cells in the publication mentioned above [20]. Similarly, in another scRNAseq study focusing on corticotroph PitNET, cells with a similar epithelial expression profile are characterized and termed epithelial progenitor cells [17].

In more detail, however, interesting differences are revealed. For example, we do not observe overexpression of stem cell markers such as *SOX2* and *SOX9* in the cell clusters expressing the epiDiff signature in either GoPN or LaPN. Moreover, at the gene level, there are also apparent differences between the epiDiff_GoPN and epiDiff_LaPN signatures, suggesting subtype-specific alterations within the epithelial-like cells. Nevertheless, both signatures are represented in all PitNET subtypes at bulk expression level. In addition, we identify another proliferative gene signature (ccTum_LaPN) within LaPN tumor cells, neither showing strong expression levels in other subtypes nor playing a role in the previously mentioned publications. This signature stands out due to its significant correlation to tumors resisting first-line therapeutic options or needing multi-therapeutical approaches, which we do not see in the other ITH signatures.

In the following steps, we concentrated on the apparent differences in the TME between GoPN and LaPN. Firstly, we confirm the higher abundance of lymphocytes in F-PitNET compared to NF-PitNET observed in previous studies [13, 48–50] and secondly, discover proliferative cells with strong correlations to aggressive and invasive tumor behavior. In recent scRNA-seq publications on PitNET, a thorough analysis of the immunological TME was sparsely done or yielded no significant results [17, 20]. However, Zhang et al. detected a proliferative cell population within corticotroph PitNET [17]. Proliferation markers included in the signature, like *Ki67* and *PTTG1,* have long been used as predictor for tumor recurrence or as invasiveness-related biomarkers in PitNET and other entities, although their value accurately predicting PitNET tumorigenesis has not been definitely confirmed [29, 51–53]. In nearly a dozen tumor entities, an overexpression of *PTTG1* is associated with a poor overall survival, and in PitNET, a correlation with higher invasiveness has been confirmed [29, 53]. Both interaction analysis and single-cell spatial transcriptomics could be valuable tools to further illuminate the involvement of proliferative tumor cells in the aggressive growth patterns of PitNET.

Our data further revealed a positive correlation between the abundance of CD8^+^ and NK lymphocytes and less aggressive, less resistant, and in the case of CD8^+^, also less invasive PitNET phenotypes. Concerning CD8^+^ T cells, this is consistent with other findings within the PIT-1 PitNET lineage. In somatotroph PitNET, a low number of CD8^+^ lymphocytes were associated with higher invasion and resistance to treatment [46, 50, 54]. Concerning NK cell infiltration in PitNET, the knowledge is limited. Although our results align with findings from CD3^−^CD56^+^ NK cells showing low abundance in invasive NF-PitNET [55], supplementary research is needed to determine the role of NK cells in the development of aggressive tumor behavior.

We consider the NK cells collectively in our data, but we notice a fragmentation of NK cells into two independent clusters in the lymphoid UMAP. In cluster 8, we observe typical NCAM^−^FCGR3A^+^ (also known as CD56^dim^CD16^+^) NK cells with increased expression levels of granzymes and perforins. In contrast, the NK cells from cluster 4 are NCAM^+^FCGR3A^−^ and show lower expression of the abovementioned cytotoxicity markers. It is reasonable to assume that these are the physiologically much rarer CD56^bright^CD16^−^ NK cells with low lytic capacity [56, 57]. In other entities, inverse correlations were found between high abundance of CD56^bright^CD16^−^ NK cells and overall survival [58]. Here, a pro-tumoral effect of CD56^bright^CD16^−^ NK cells through inhibition of T cell responses via *CD38*, *perforin*, *CD11a,* and *IFNγ*, was postulated. The NCAM^−^FCGR3A^+^ NK cells (cl. 8) with higher cytotoxic capacity are distributed equally in number between GoPN and LaPN in our data. In contrast, it is striking that the NCAM^+^FCGR3A^−^ NK cells (cl. 4) with reduced lytic capacity are, in the majority, attributable to LaPN, the more aggressive PitNET subtype. This suggests that NCAM^+^FCGR3A^−^NK cells may exert a pro-tumoral effect in LaPN and, therefore may be targets of future cancer immunotherapies in aggressive LaPN.

Further, we would like to point out the observation of a population of CD4^−^/CD8^−^ double negative lymphocytes, the largest lymphoid fraction in our LaPN dataset. To the best of our knowledge, we are the first to report this finding in the TME of F-PitNET. Double negative CD3^+^ T cells (referred to in the literature as DN T cells) represent 1–3% of mature peripheral T cells, and both residential as well as organ- and tumor-infiltrating DN T cells have been observed over the years [40]. Despite increasing scientific interest, their role in tumorigenesis is not yet sufficiently deciphered. Their occurrence is associated with both pro- and anti-tumor effects, depending on the tumor type and their distinct TME. After observing a significant increase of DN T cells in lymph nodes of melanoma patients with recent disease progression, a contribution of DN T cells to tumor metastatic progression was hypothesized [40]. Our data does not reveal any significant correlation between the abundance of CD4^−^/CD8^−^ DN T cells and invasive growth patterns in F-PitNET. The reasons for this may be manifold and leave us to speculate about the exact function of these cells in the TME of F-PitNET. However, due to their massive presence in LaPN and complete absence in GoPN (and perhaps other PitNET subtypes), they should be considered a potentially valuable target for cancer immunotherapy in drug-resistant LaPN.

## Conclusion

In our comparative study of the cellular composition of gonadotroph and lactotroph PitNET, we identify proliferative cells within tumor cells of lactotroph PitNET correlating with high aggressiveness in a larger cohort. In addition, detailed analysis of the lymphoid cell compartment within both PitNET subtypes reveals proliferative lymphocytes with a strong correlation to aggressive and invasive tumor behavior.

Our results lay the foundation for further studies on the influence of lymphoid cell components on the variable aggressive behavior of PitNET. They point out potential targets for cancer immunotherapy and therefore may pave the way for the development of new therapeutic options.

## Supplementary Information

Supplementary Material 1: Fig. 1 (related to Figure 1). UMAP plots show the origin and distribution of single-cell transcriptomes from the individual GoPN (a) or LaPN (b) sample.

Supplementary Material 2: Fig. 2 (related to Figure 1). Dot plots show the average expression level (avg. exp.) and occurrence (pct. exp.) of cell type-specific marker genes in clusters of GoPN (a) and LaPN (b).

Supplementary Material 3: Fig. 3 (related to Figure 1). Inferred CNV analysis of GoPN (a) and LaPN (b) samples, sorted by cluster (top bar) and affiliation to tumor or TME compartments (dashed vertical line). (c) Expression of selected endocrine marker genes in the tumor cell clusters (red) and TME cluster (black) of LaPN only.

Supplementary Material 4: Fig. 4 (related to Figure 2). (a) Relative proportion of PitNET samples (n = 134) from Neou et al. bulk RNA-seq cohort divided by aggressiveness levels (*Rem*, remission; *Pers*, persistent; *Res*, resistant; *Agg*, aggressive). *LaPN *(n = 16); *PluriPN* (n = 9); *CorPN* (n = 35); *ThyPN* (n = 6); *SomPN* (n = 23); *NCPN* (n = 8); *GoPN* (n = 29); *MixedPN* (n = 8).

Supplementary Material 5: Fig. 5 (related to Figure 3). (a) Relative proportion of GoPN samples (n = 7) within the clusters of the TME. (b) Relative proportion of LaPN samples (n = 3) within the clusters of the TME.

Supplementary Material 6: Fig. 6 (related to Figure 4). (a) Percentages of myeloid cells from individual GoPN and LaPN samples in each cluster. (b) Feature plots of highly specific marker genes for annotated cell types.

Supplementary Material 7: Fig. 7 (related to Figure 5). (a) Percentages of lymphoid cells from individual GoPN and LaPN samples in each cluster. (b) Average expression levels (avg. exp.) and occurrence (pct. exp.) of cell type-specific marker genes in the different clusters.

Supplementary Material 8: Fig. 8 (related to Figure 5). Correlation of lymphoid cell type signatures derived from GoPN/LaPN single-cell data with the degree of aggressiveness of PitNET tumors in a bulk RNA-seq cohort [28]. Statistical analysis was performed using an unpaired two-sample Wilcoxon test, with */**/***/**** indicating *p* ≤ 0.05/0.01/0.001/0.0001, respectively. Non-significant results remain unmarked.

Supplementary Material 9.

Supplementary Material 10.

Supplementary Material 11.

Supplementary Material 12.

Supplementary Material 13.

Supplementary Material 14.

Supplementary Material 15.

Supplementary Material 16.

Supplementary Material 17.

## Data Availability

The single-cell RNA sequencing data generated in this study have been deposited in NCBI’s Gene Expression Omnibus and are accessible through GEO accession number GSE244101.

## Abbreviations

ACTH: Adrenocorticotropic hormones
act-Macs: Activated macrophages
Agg: Aggressive
APG: Anterior pituitary gland
CCNB1: Cyclin B1
ccTum: Cycling tumor cells
cDC: *CD1C*-expressing conventional dendritic cells
CENPF: Centromere protein F
CHGA: Chromogranin A
CNV: Copy number variation
CorPN: Corticotroph pituitary neuroendocrine tumors
CPE: Carboxypeptidase E
DEG: Differentially expressed genes
DN T cells: Double negative CD3^+^ T cells
EC: Endothelial cells
epiDiff: Epithelial differentiation and proliferation
F-PitNET: Functioning pituitary neuroendocrine tumors
FSH: Follicle-stimulating hormone
GH: Growth hormone
GO: Gene Ontology
GoPN: Gonadotroph pituitary neuroendocrine tumors
hormSec: Hormone secretion
IL1B: Interleukin 1 beta
ILC: Innate lymphoid cells
ITH: Intratumoral heterogeneity
LaPN: Lactotroph pituitary neuroendocrine tumors
LH: Luteinizing hormone
M2-Macs: M2-polarized macrophages
MC: Mast cells
MDSC: Myeloid-derived suppressor cells
MHC: Major histocompatibility complex
MiMe: Mitotic and metabolic genes
MixedPN: Mixed pituitary neuroendocrine tumors
MKI67: Proliferation marker Ki-67
MP: Metaprograms
Mono: Monocytes
NCPN: Null-cell pituitary neuroendocrine tumors
NF-PitNET: Non-functioning pituitary neuroendocrine tumors
NK: Natural killer
NKT: Natural killer T
NR5A1: Nuclear receptor subfamily 5 group A member 1
PC: Pericytes
Pers: Persistent
PitNET: Pituitary neuroendocrine tumors
Plasma: Plasma B cells
PluriPN: Plurihormonal pituitary neuroendocrine tumors
POU1F1: POU class 1 homeobox 1
PRL: Prolactin
Rem: Remission
Res: Resistant
SCG5: Secretogranin V
scRNA-seq: Single-cell RNA sequencing
SomPN: Somatotroph pituitary neuroendocrine tumors
TAMs: Tumor-associated macrophages
TFs: Transcription factors
ThyPN: Thyrotroph pituitary neuroendocrine tumors
TME: Tumor microenvironment
TPIT: T-box transcription factor 19
TSH: Thyroid-stimulating hormone
UMAP: Uniform Manifold Approximation and Projection
WHO: World Health Organization
