# Supplementary figures and images for: Single-cell transcriptomics link gene expression signatures to clinicopathological features of gonadotroph and lactotroph PitNET
Source: J Transl Med. 2024 Nov 15;22:1027. doi: 10.1186/s12967-024-05821-4 (PMC11566263; doi:10.1186/s12967-024-05821-4)

**a**

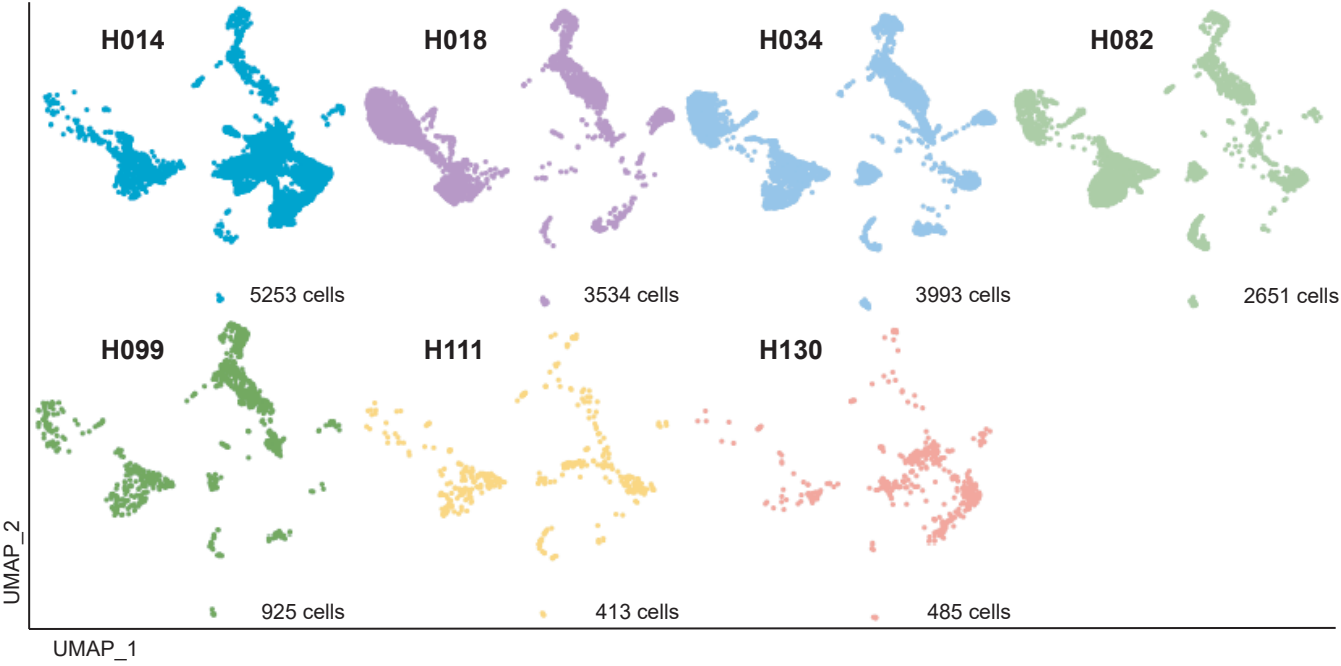

**b**

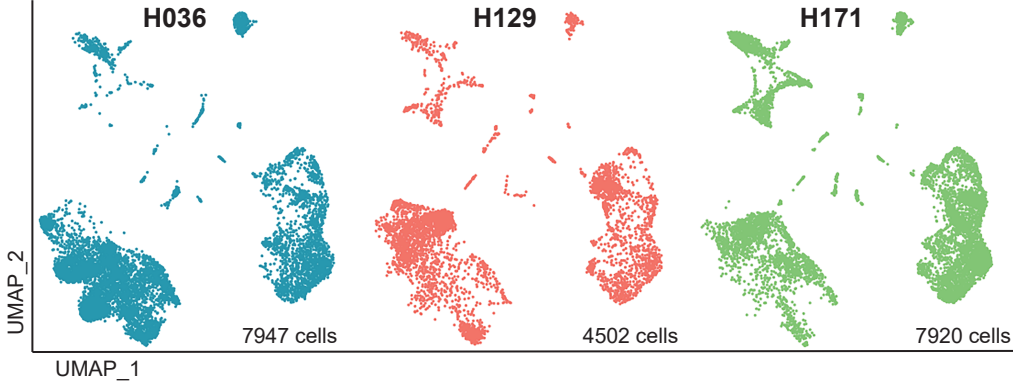

Supplement: Supplementary file 1 — Supplementary Material 1: Fig. 1 (related to Figure 1). UMAP plots show the origin and distribution of single-cell transcriptomes from the individual GoPN (a) or LaPN (b) sample. [file 12967_2024_5821_MOESM1_ESM.pdf]

**a****GoPN**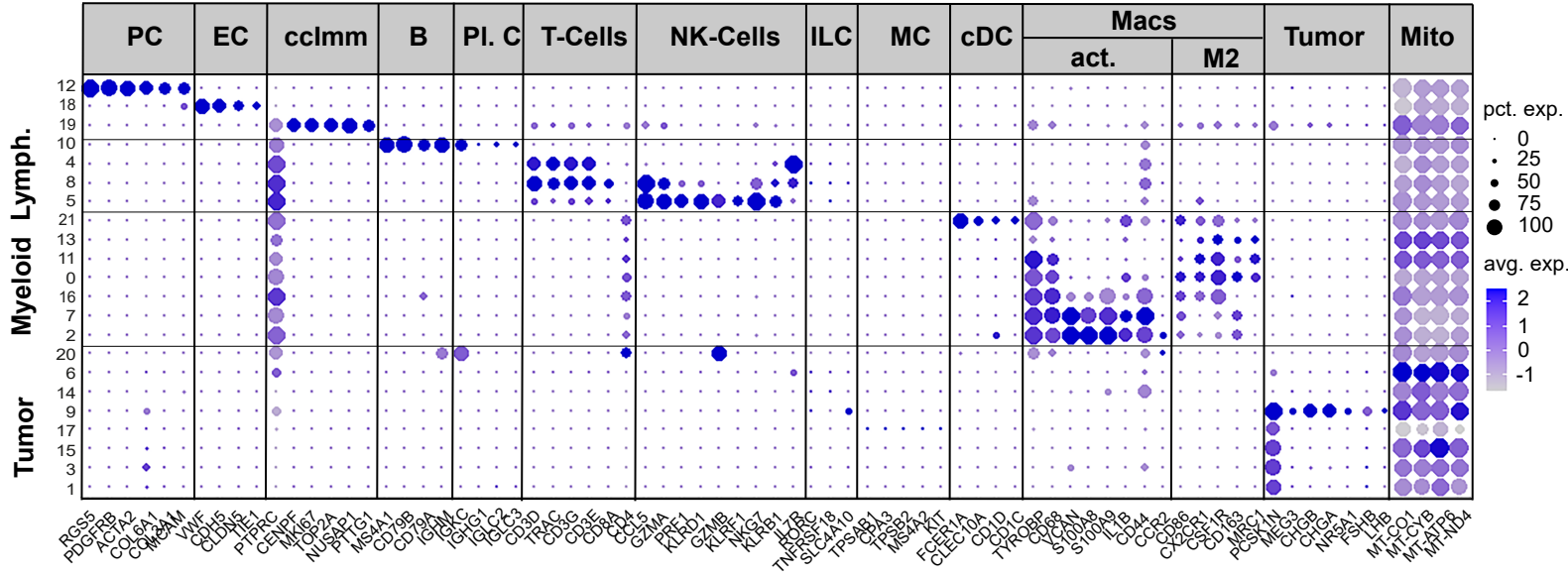**b****LaPN**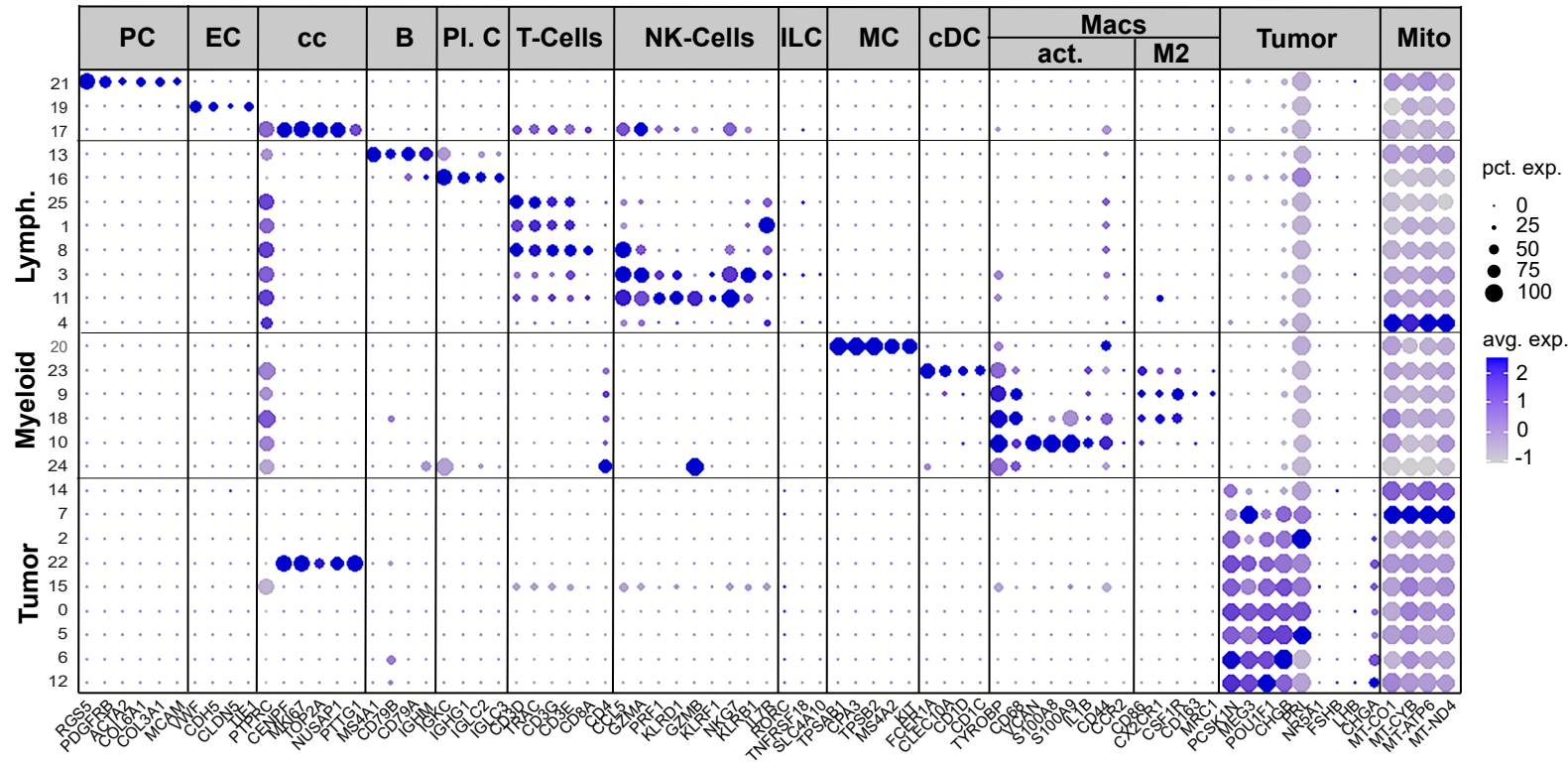

Supplement: Supplementary file 2 — Supplementary Material 2: Fig. 2 (related to Figure 1). Dot plots show the average expression level (avg. exp.) and occurrence (pct. exp.) of cell type-specific marker genes in clusters of GoPN (a) and LaPN (b). [file 12967_2024_5821_MOESM2_ESM.pdf]

**a**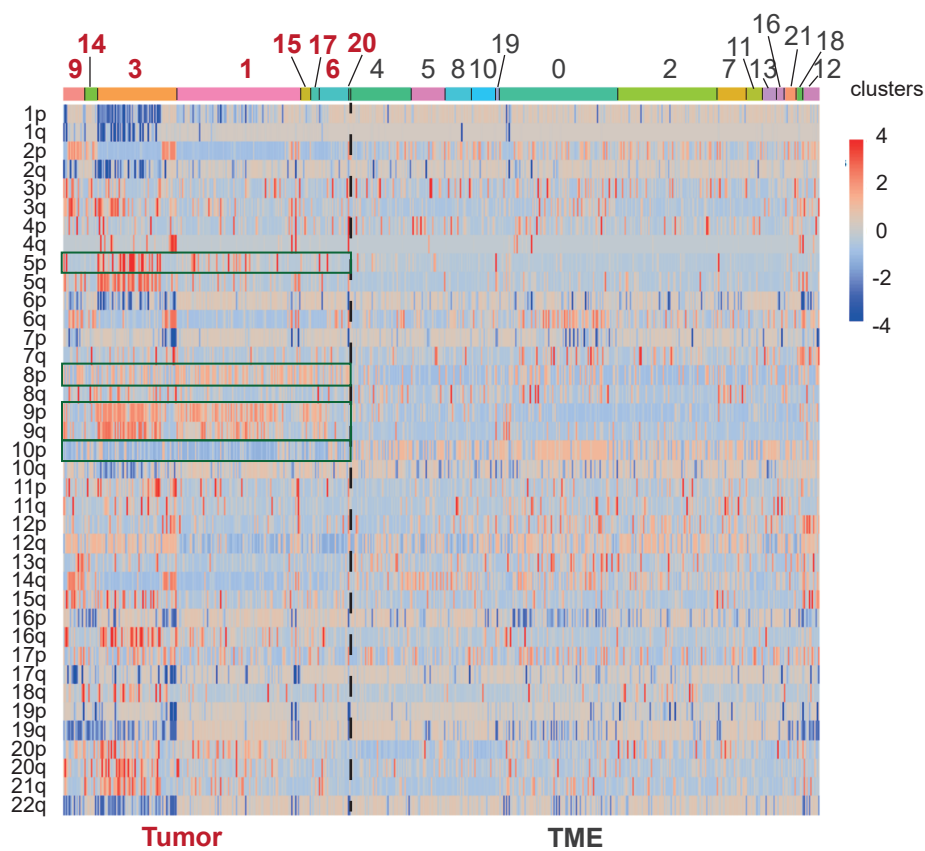**b**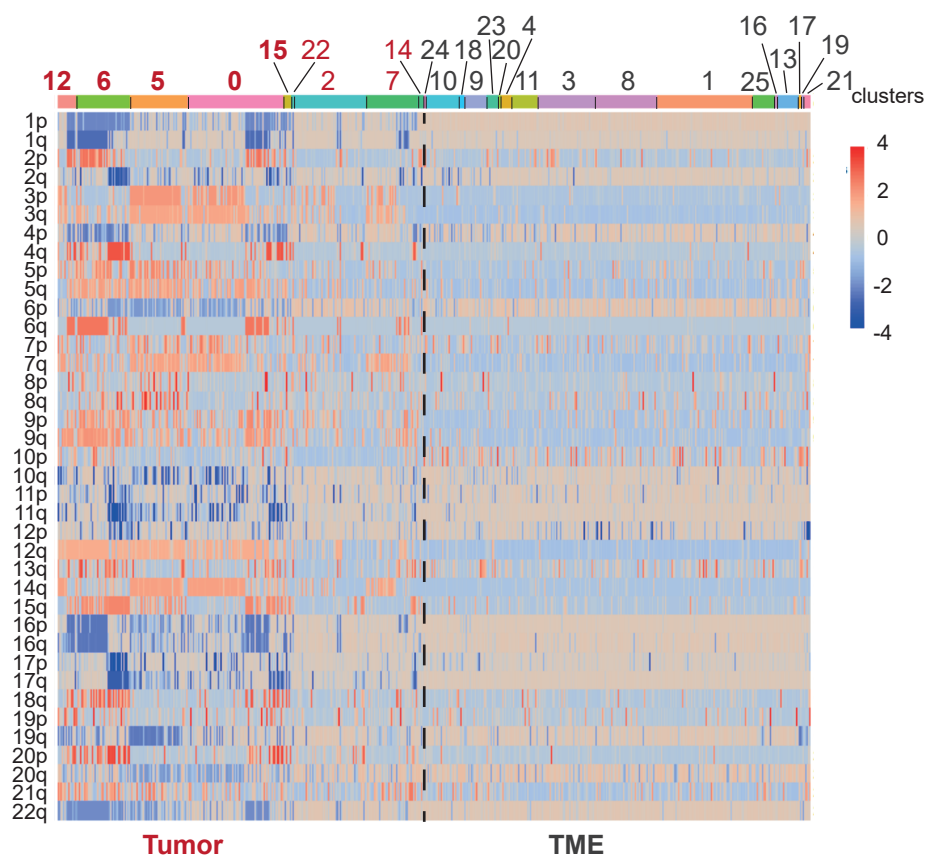**c**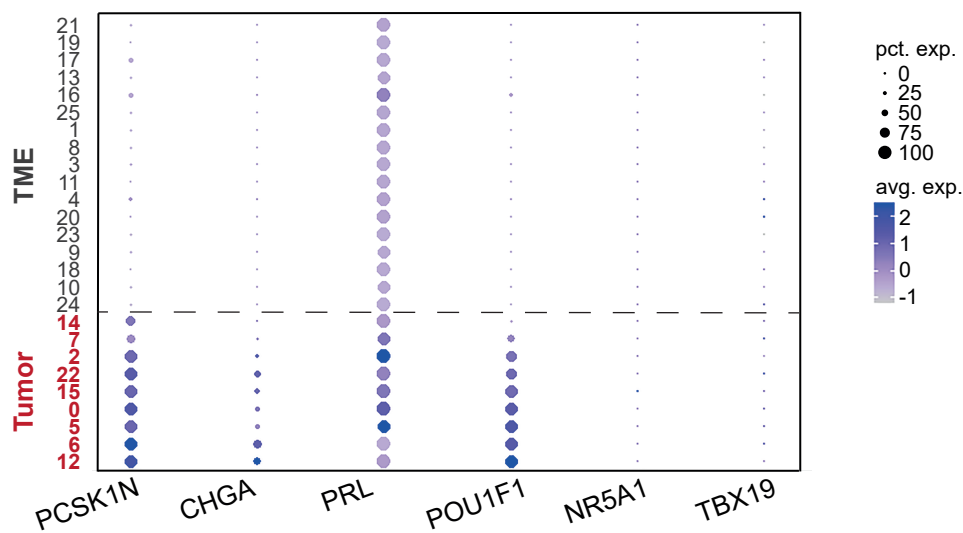

Supplement: Supplementary file 3 — Supplementary Material 3: Fig. 3 (related to Figure 1). Inferred CNV analysis of GoPN (a) and LaPN (b) samples, sorted by cluster (top bar) and affiliation to tumor or TME compartments (dashed vertical line). (c) Expression of selected endocrine marker genes in the tumor cell clusters (red) and TME cluster (black) of LaPN only. [file 12967_2024_5821_MOESM3_ESM.pdf]

**a**

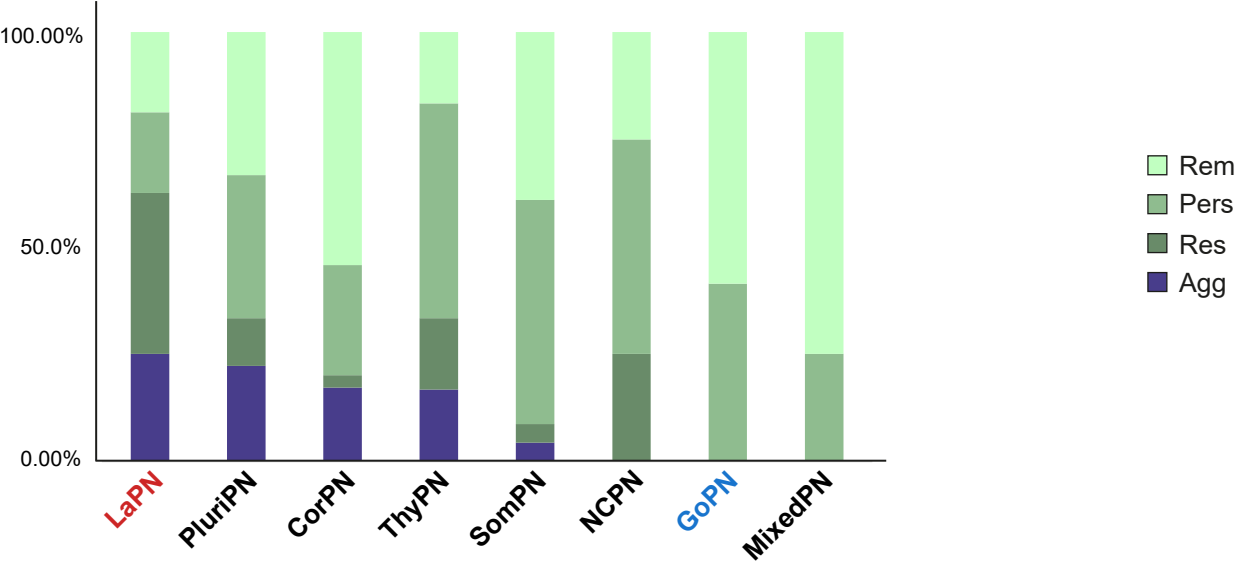

Supplement: Supplementary file 4 — Supplementary Material 4: Fig. 4 (related to Figure 2). (a) Relative proportion of PitNET samples (n = 134) from Neou et al. bulk RNA-seq cohort divided by aggressiveness levels (Rem, remission; Pers, persistent; Res, resistant; Agg, aggressive). LaPN (n = 16); PluriPN (n = 9); CorPN (n = 35); ThyPN (n = 6); SomPN (n = 23); NCPN (n = 8); GoPN (n = 29); MixedPN (n = 8). [file 12967_2024_5821_MOESM4_ESM.pdf]

**a**

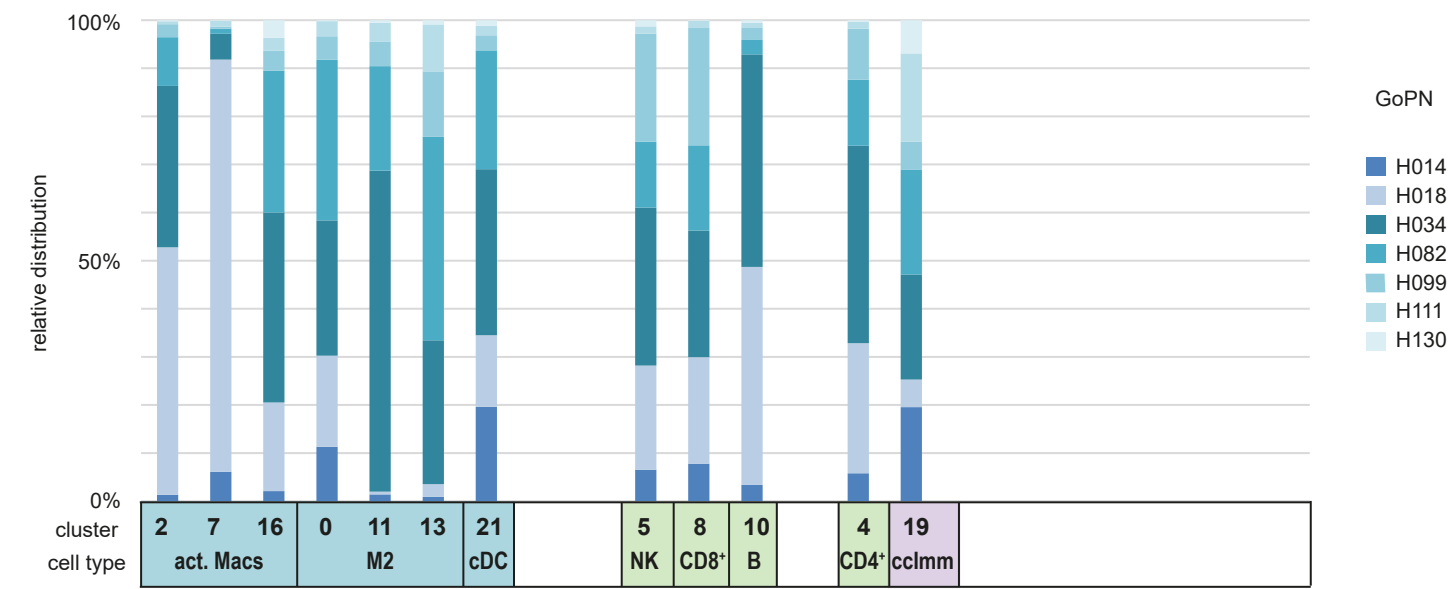

**b**

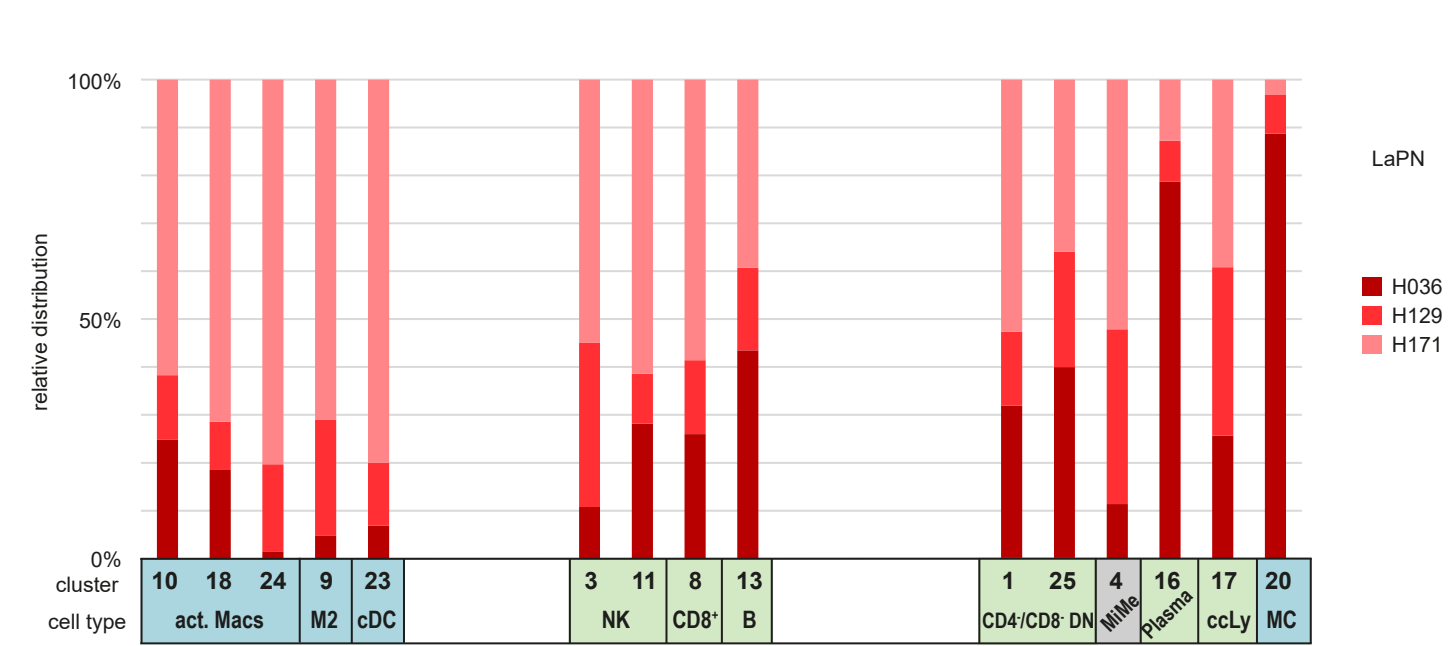

Supplement: Supplementary file 5 — Supplementary Material 5: Fig. 5 (related to Figure 3). (a) Relative proportion of GoPN samples (n = 7) within the clusters of the TME. (b) Relative proportion of LaPN samples (n = 3) within the clusters of the TME. [file 12967_2024_5821_MOESM5_ESM.pdf]

**a**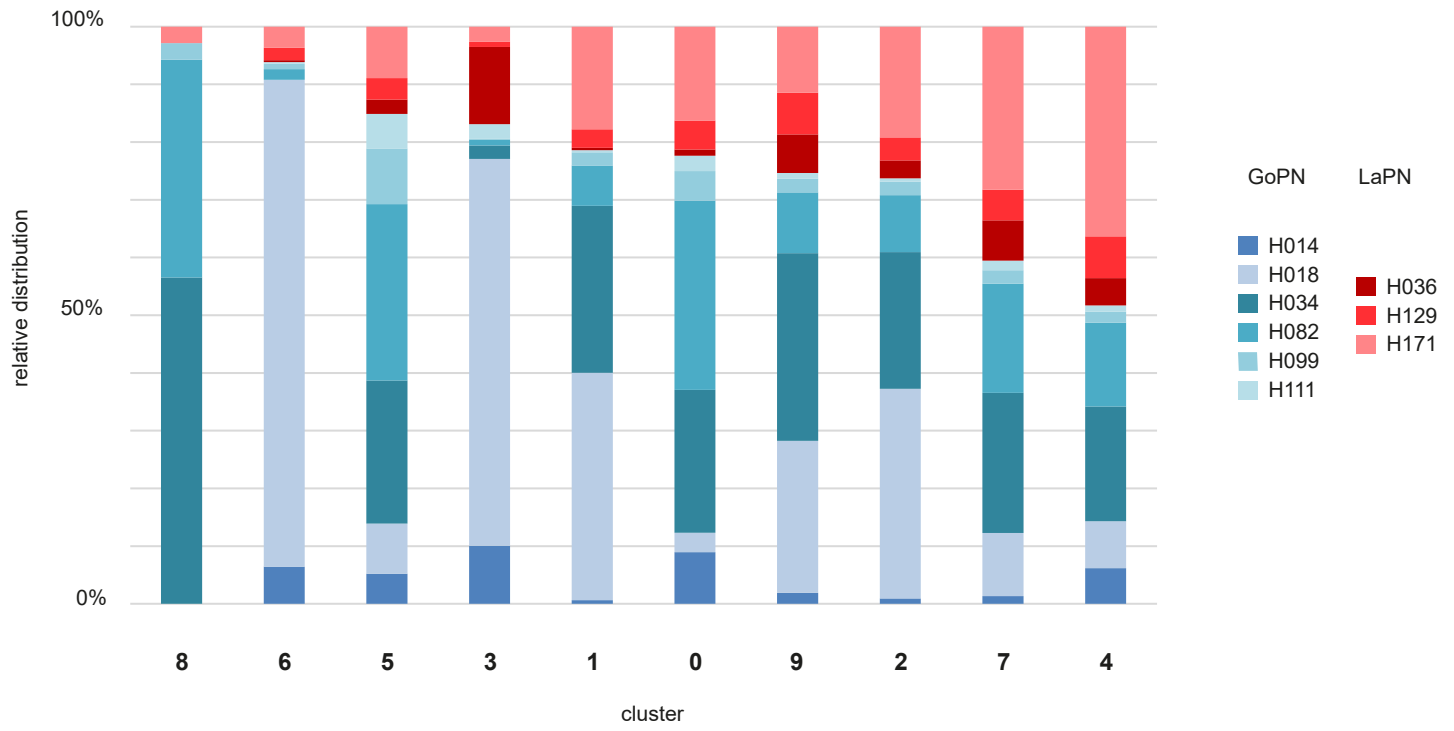**b**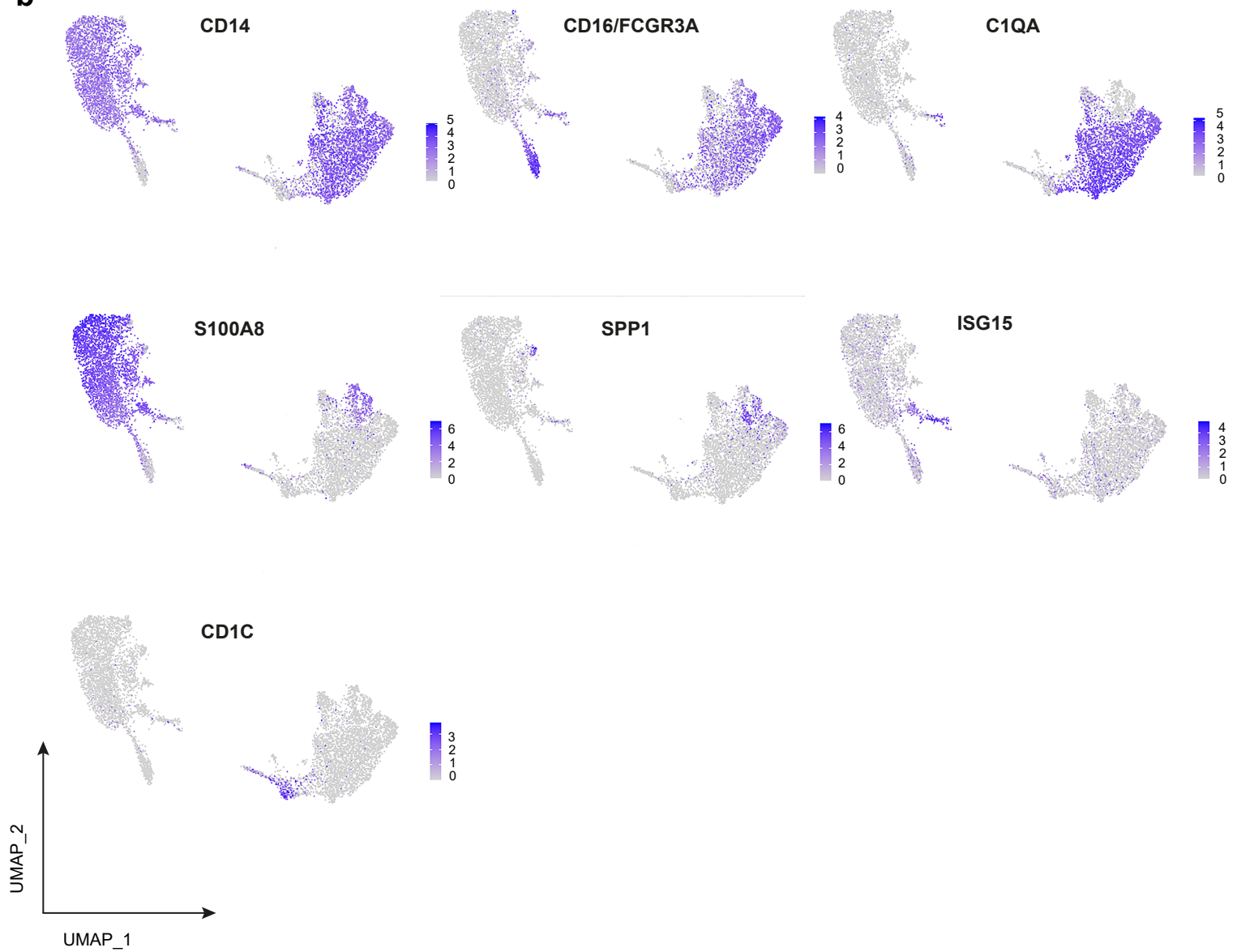

Supplement: Supplementary file 6 — Supplementary Material 6: Fig. 6 (related to Figure 4). (a) Percentages of myeloid cells from individual GoPN and LaPN samples in each cluster. (b) Feature plots of highly specific marker genes for annotated cell types. [file 12967_2024_5821_MOESM6_ESM.pdf]

a

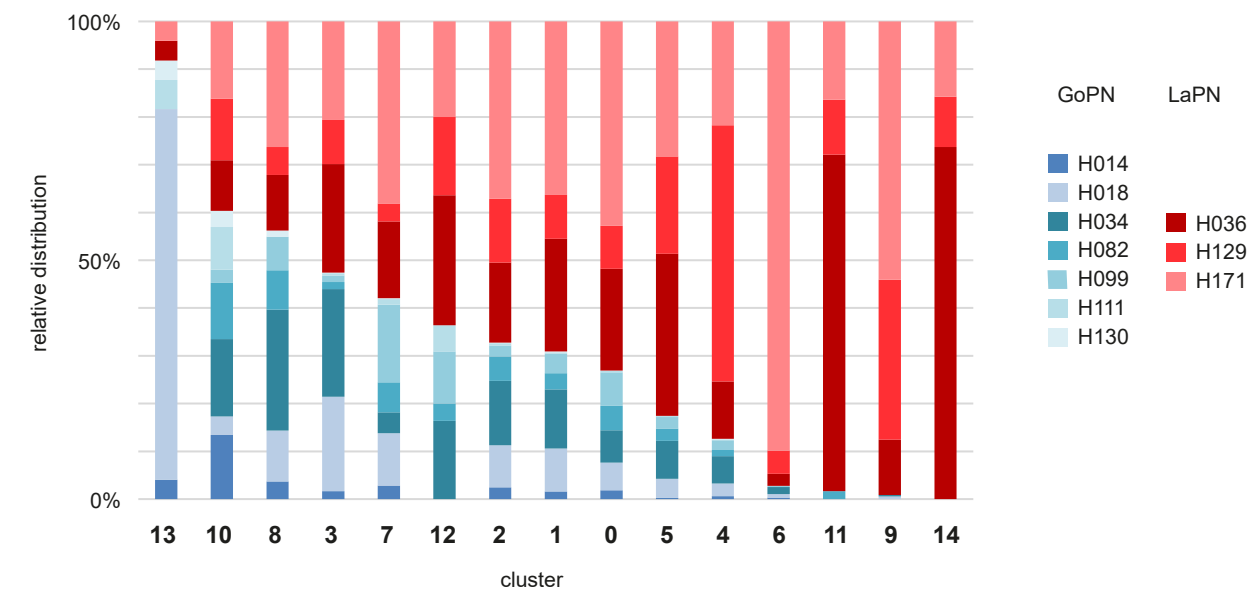

b

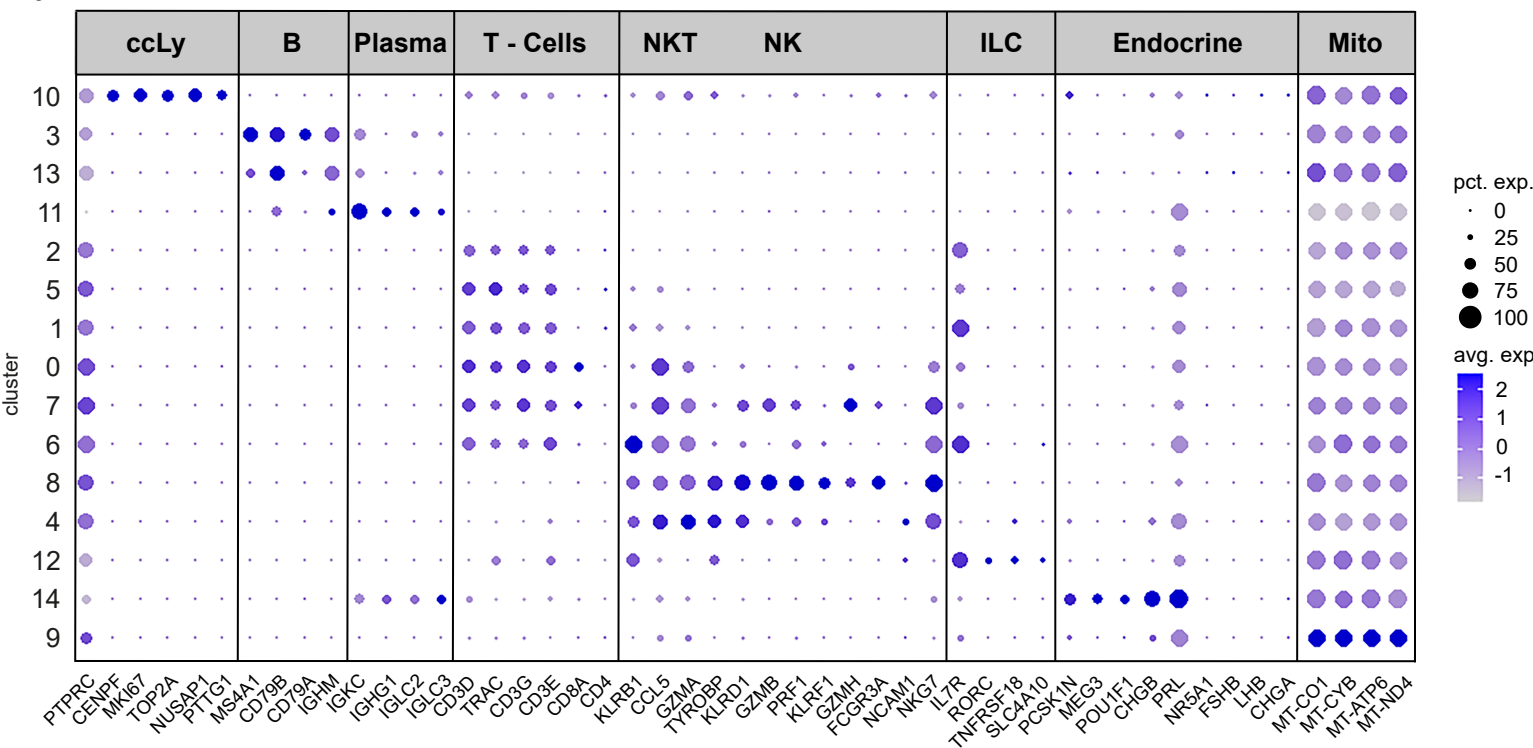

Supplement: Supplementary file 7 — Supplementary Material 7: Fig. 7 (related to Figure 5). (a) Percentages of lymphoid cells from individual GoPN and LaPN samples in each cluster. (b) Average expression levels (avg. exp.) and occurrence (pct. exp.) of cell type-specific marker genes in the different clusters. [file 12967_2024_5821_MOESM7_ESM.pdf]

**a**      Aggressiveness

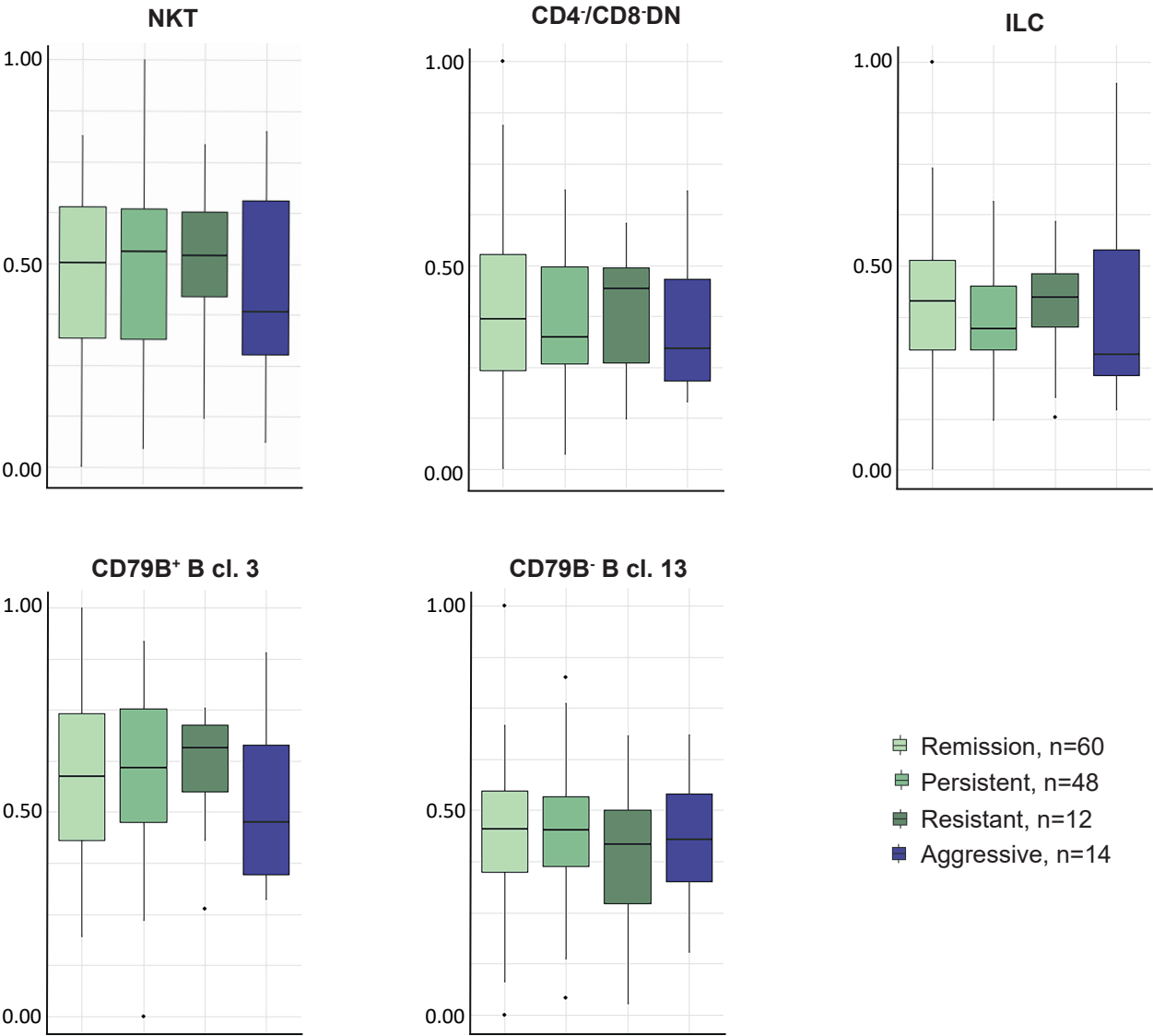

Supplement: Supplementary file 8 — Supplementary Material 8: Fig. 8 (related to Figure 5). Correlation of lymphoid cell type signatures derived from GoPN/LaPN single-cell data with the degree of aggressiveness of PitNET tumors in a bulk RNA-seq cohort [28]. Statistical analysis was performed using an unpaired two-sample Wilcoxon test, with */**/***/**** indicating p ≤ 0.05/0.01/0.001/0.0001, respectively. Non-significant results remain unmarked. [file 12967_2024_5821_MOESM8_ESM.pdf]
